# Supplementary material for: Accelerating glacier volume loss on Juneau Icefield driven by hypsometry and melt-accelerating feedbacks
Source: Nat Commun. 2024 Jul 2;15:5099. doi: 10.1038/s41467-024-49269-y (PMC11220083; doi:10.1038/s41467-024-49269-y)
Supplement: Supplementary file 1 — Supplementary Information [file 41467_2024_49269_MOESM1_ESM.pdf]

Accelerating glacier volume loss on Juneau Icefield driven by hypsometry and melt-accelerating feedbacks: Supplementary Information

Bethan Davies, Robert McNabb, Jacob Bendle, Jonathan Carrivick, Jeremy Ely, Tom Holt, Bradley Markle, Christopher McNeil, Lindsey Nicholson, Mauri Pelto

1 CONTENTS

2 Juneau Icefield glaciology and climate 2
3 The “Little Ice Age” 7
3.1 Climate during the “Little Ice Age” 7
3.2 Evidence for glaciation in Alaska during the ‘Little Ice Age’ 7
4 Supplementary Methods 10
4.1 Data sources, 1948 and 1979 10
4.2 Assessment of Topographic Maps 13
4.3 Source and date of satellite imagery, 1980s onwards 16
4.4 Analysis of uncertainty in glacier area 17
5 Supplementary Results 19
5.1 Little Ice Age glaciation 19
5.2 Glacier change, LIA to 1948 24
5.3 Glacier change, 1948 - 1979 26
5.4 Glacier change, 1979 – 2005 26
5.5 Glacier change, 2005-2019 27
5.6 Glacier snowlines 30
5.7 Albedo 35
5.7.1 All sensors 35
5.7.2 Landsat 5 and 7 only 39
6 References 40

2 JUNEAU ICEFIELD GLACIOLOGY AND CLIMATE

Supplementary table 1. Mean temperatures at Juneau Airport meteorological station calculated from ref. [1]. The 1986-2005 mean is provided for straightforward comparison with the IPCC Assessment Report 6[2].

	Mean annual temperatures (°C)	Mean summer temperatures (°C)	Mean winter temperatures (°C)
**1941-1970**	4.54	12.37	-3.14
**1971-2000**	5.27	13.01	-2.41
**2001-2020**	5.67	13.34	-1.07
**1986-2005**	5.78	13.39	-1.22

Supplementary table 2. Climatic data for each period from the Juneau meteorological station[1]. Summer temperature anomaly is compared with the 1986-2005 AD mean.

Year (AD)	Mean annual air temperature (°C)	Summer temperature anomaly (°C)	Mean winter accumulation (m w.e.)
1770			
1941-1948	5.12	-0.83	4.67
1949-1979	4.35	-1.02	10.27
1980-1990	5.57	-0.30	11.39
1991-2005	5.77	0.01	14.27
2006-2015	5.32	-0.40	13.59
2016-2019	6.17	0.55	16.48
1986-2005	5.78		

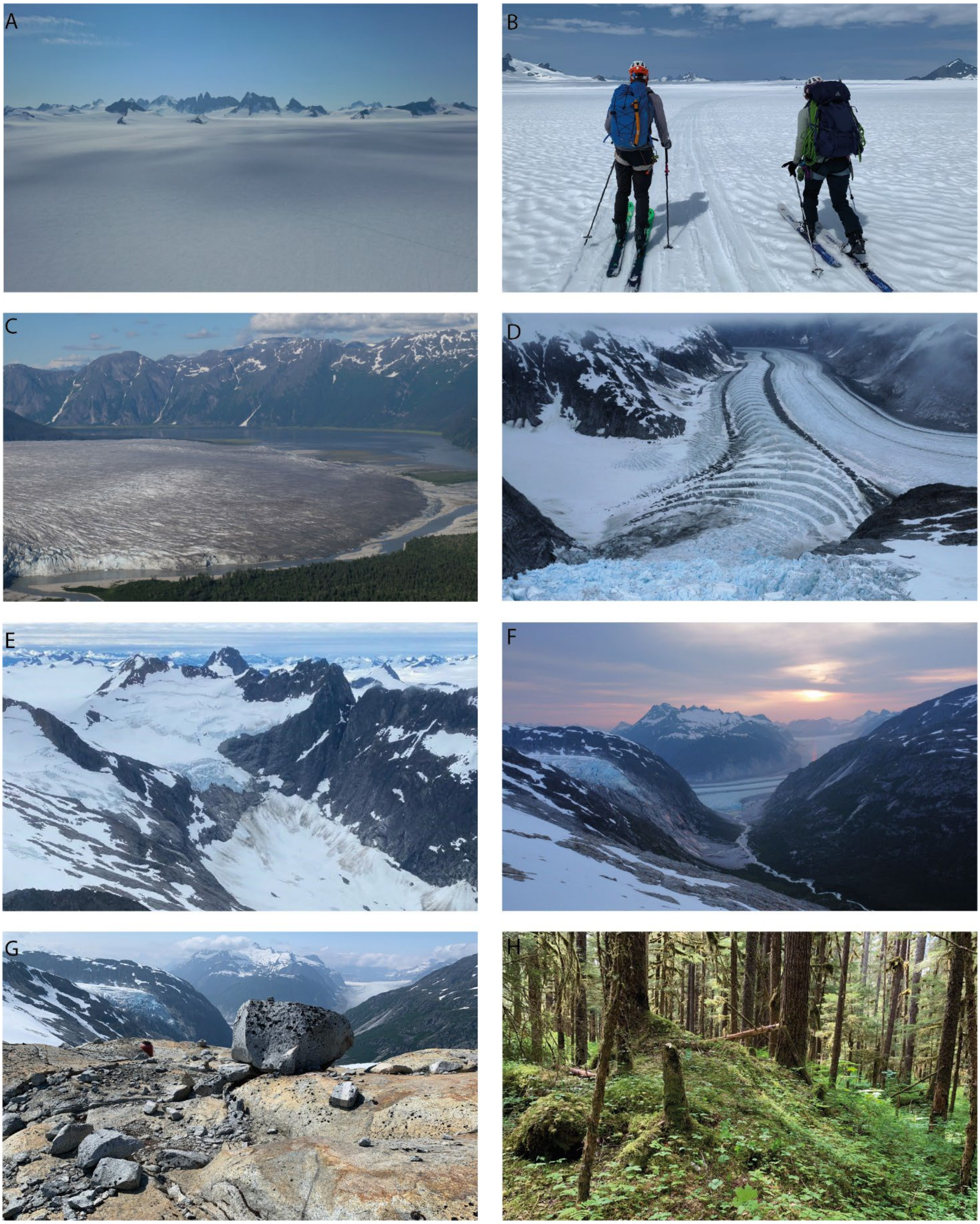

Supplementary figure 1. Field photographs of Juneau Icefield, all from July 2022. A, B: the low-slope plateau accumulation area of Taku Glacier. C: The piedmont terminus of Taku Glacier, showing the shoaling moraines building up. D: Looking down Gilkey Glacier, with ogives on the tongue, and the icefall in the foreground. E: Fragmentation of a small glacier on the icefield. F: Avalanche Canyon, a deep, ice-scoured valley, with Gilkey Glacier in the background. G: recently exposed ice-scored bedrock at high elevations above Avalanche Canyon. H: Neoglacial terminal moraines in the Herbert Glacier forefield. All photographs credit BJD.

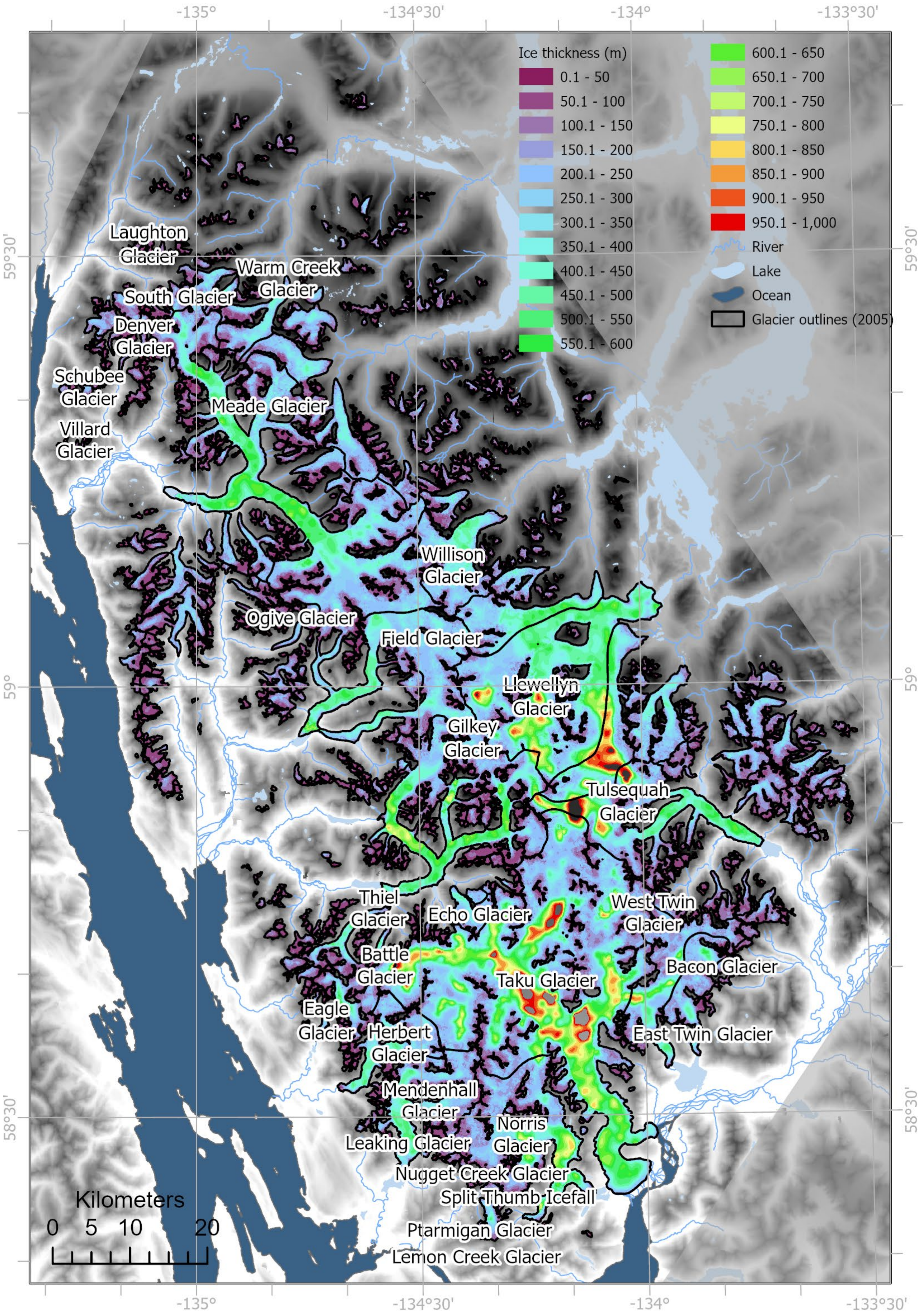

Supplementary figure 2. Ice thicknesses of Juneau Icefield. From data published in Ref.[3]. Overlain on ASTER GDEM, the Global Digital Elevation Model produced by ASTER, courtesy of NASA/JPL-Caltech.

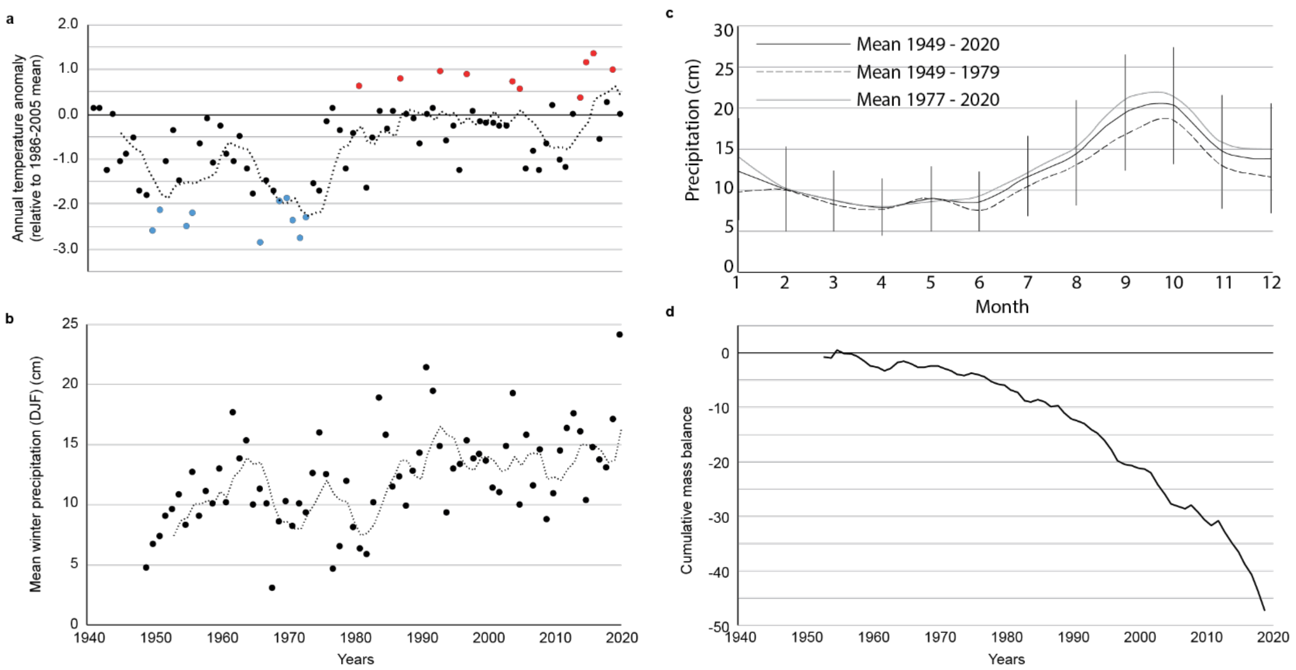

Supplementary figure 3. a-c: Meteorological data from Juneau meteorological station at Juneau Airport (NOAA; https://www.ncdc.noaa.gov/cag/city/time-series/USW00025309). a. Annual temperature anomaly compared with the 1986-2005 mean. Ten warmest (red) and ten coldest (blue) years are indicated. b. Mean winter precipitation (December, January, February; DJF) at Juneau meteorological station from 1948 to 2021, with a 5-year moving average. c. Monthly mean precipitation values for Juneau Airport meteorological station, 1949-2020, 1949-1975 and 1977-2020. Error bars are one standard deviation of the 1949 – 2020 mean. d. Glacier mass balance, Lemon Creek Glacier, 1953 – 2019, from Ref. [4].

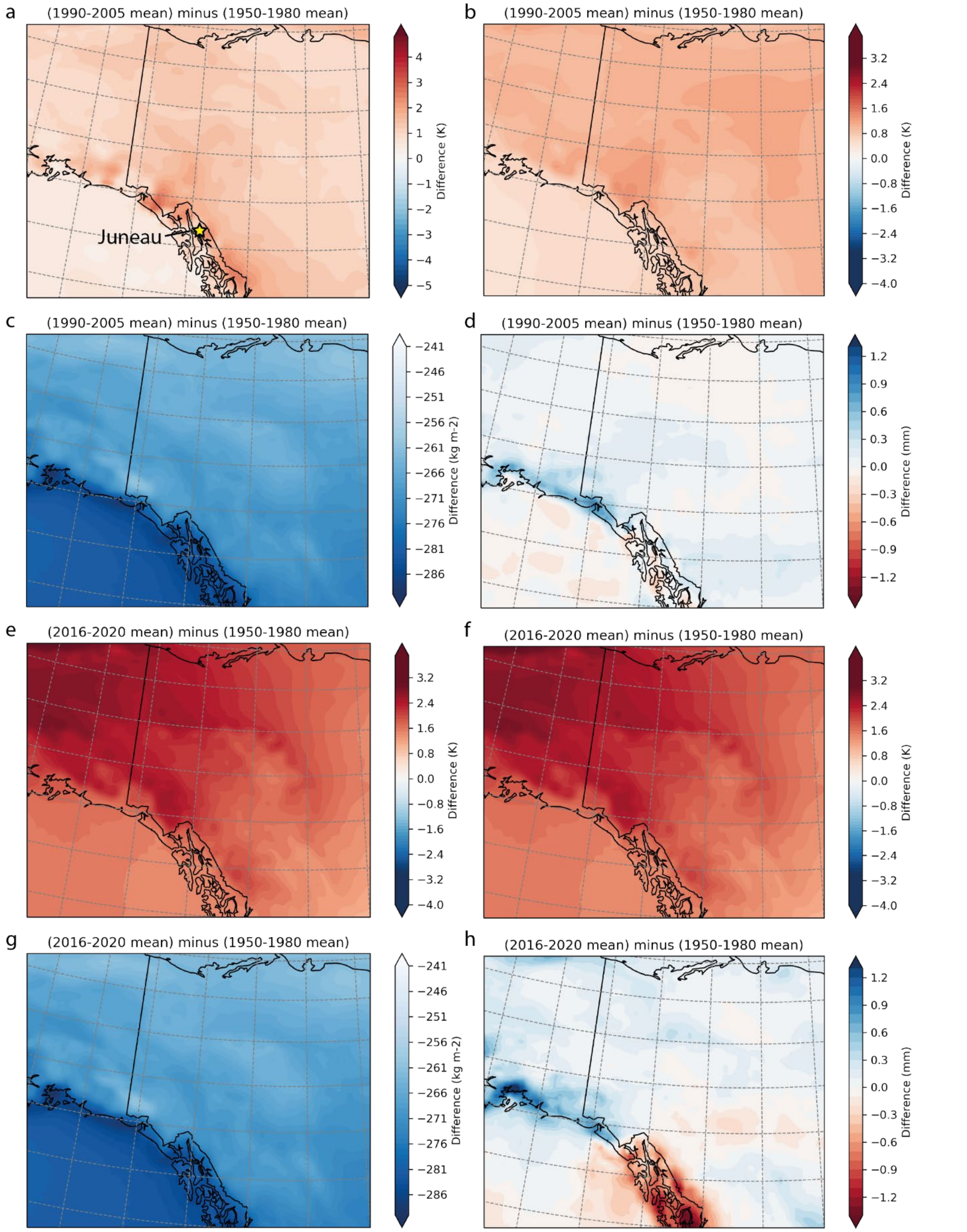

Supplementary figure 4. ERA5 climate reanalysis data[5] for NW North America. Location of Juneau is shown with yellow star in panel a. A-d show the grid cell difference from 1990-2005 mean versus the 1950-1980 mean. E-h show the difference for the 2016-2020 mean versus the 1950-1980 mean. A. t2m air temperature at 2 metres above ground level. B. t850 temperature at 850 mb (approximately 1500 m asl, close to the Juneau Icefield plateau). C. vmid vertically integrated moisture difference. D. tp total precipitation. E. t2m air temperature at 2 metres above ground level. f. t850 temperature at 850 mb (approximately 1500 m asl, close to the Juneau Icefield plateau). g. vmid vertically integrated moisture difference. h. tp total precipitation.

3 THE "LITTLE ICE AGE"

3.1 Climate during the "Little Ice Age"

In the Gulf of Alaska, the "Little Ice Age" (LIA) (1250 – 1850 AD) is well recorded in a number of palaeoclimatic proxies, including dendrochronology[6,7], coleopteran[8], and ice cores[9]. In central Alaska, at the continental-climate Farewell Lake in central Alaska, the coldest part of the late Holocene culminated at 1700 AD[10], with temperatures 1.7°C cooler than present. At Iceberg Lake, Bagley Icefield, the timing of maximum cooling is at 1650 AD and 1850 AD, when temperatures were 1°C cooler than modern[11]. In south-west Alaska, a coleopteran palaeoclimate record gives a summer temperature of 1.3°C cooler than the modern mean (1983-2016), with cooling centred on AD 1815 AD[8]. Ice core data from Mount Hunter indicate that temperatures are now 2°C warmer than the height of the LIA[9].

3.2 Evidence for glaciation in Alaska during the 'Little Ice Age'

Across the American Cordillera, there is widespread evidence of a Late Holocene Neoglaciation, often termed the "Little Ice Age" (LIA)[12-14]. This last Neoglacial advance left behind a distinctive geomorphic system of sharp-crested moraines, trimlines, ice-scoured bedrock and thicknesses of glacial sediment. These advances were frequently the most extensive of the last 10,000 years[14,15]. Most glaciers in Alaska below 1500 m asl have an uninterrupted history of continuous recession since this maximum[16].

There is long history of research and substantial evidence of a readvance of glaciers around Juneau Icefield during the LIA[17-22]. A holistic geomorphic map was recently published[23], but a reconstruction of icefield extent at this time has not yet been attempted. Around the Juneau Icefield, outlet glaciers advanced in the prior to the LIA, in the last millennium, but these advances were generally within "Little Ice Age" limits[14,24], which dated to ca. 1750-1770 in this area[21,25]. The timing of glacier recession was largely synchronous across the icefield, with recession beginning for most glaciers between AD 1750 and 1785[17,25]. Local relative sea level data indicate that larger-than-present glaciers had stabilised by the mid-16th century, with land first emerging as glaciers shrank between AD 1770 – 1790[25]. Glaciers have been consistently shrinking since this maximum[18], with the exception of Taku Glacier, the largest outlet glacier of Juneau Icefield, which has been shrinking since 2018[26].

Taku Glacier (Supplementary figures 1C, 5) reached its maximum position three miles beyond the 1948 terminus, near Taku Point, in AD 1750[27] to AD 1755[17], where it was confluent with Norris Glacier[17,28,29]. These dendrochronological ages agree with radiocarbon ages from nearby Loon Bog[27]. At this time, Taku Glacier blocked the end of Taku Inlet[18], calving into the fjord, and dammed Taku River, causing an ice-dammed lake to form behind the glacier snout[17,27]. By 1794 AD, Taku Glacier had already receded across Taku River[27] and Taku Inlet was ice-free for most of the 19th century[18]. It was surveyed in 1890 AD by the United States Coast and Geodetic Survey, and was well inland of the 1948 position then, calving into Taku Inlet[17]. Taku Glacier advanced to a new position at the mouth of the valley by 1948, and then was then slowly advancing for most of the twentieth century[26,30], advancing 7.3 km between 1890 and the early 1990s[27]. Calving into Taku Inlet decreased in the early 20th century, as a moraine shoal rose above sea level. Calving had largely ceased by 1952. The glacier is now separated from tidewater by terminal moraines and outwash deposits[27]. Taku Glacier has only recently began to recede again, with a negative mass balance recorded from AD 2013 onwards[26].

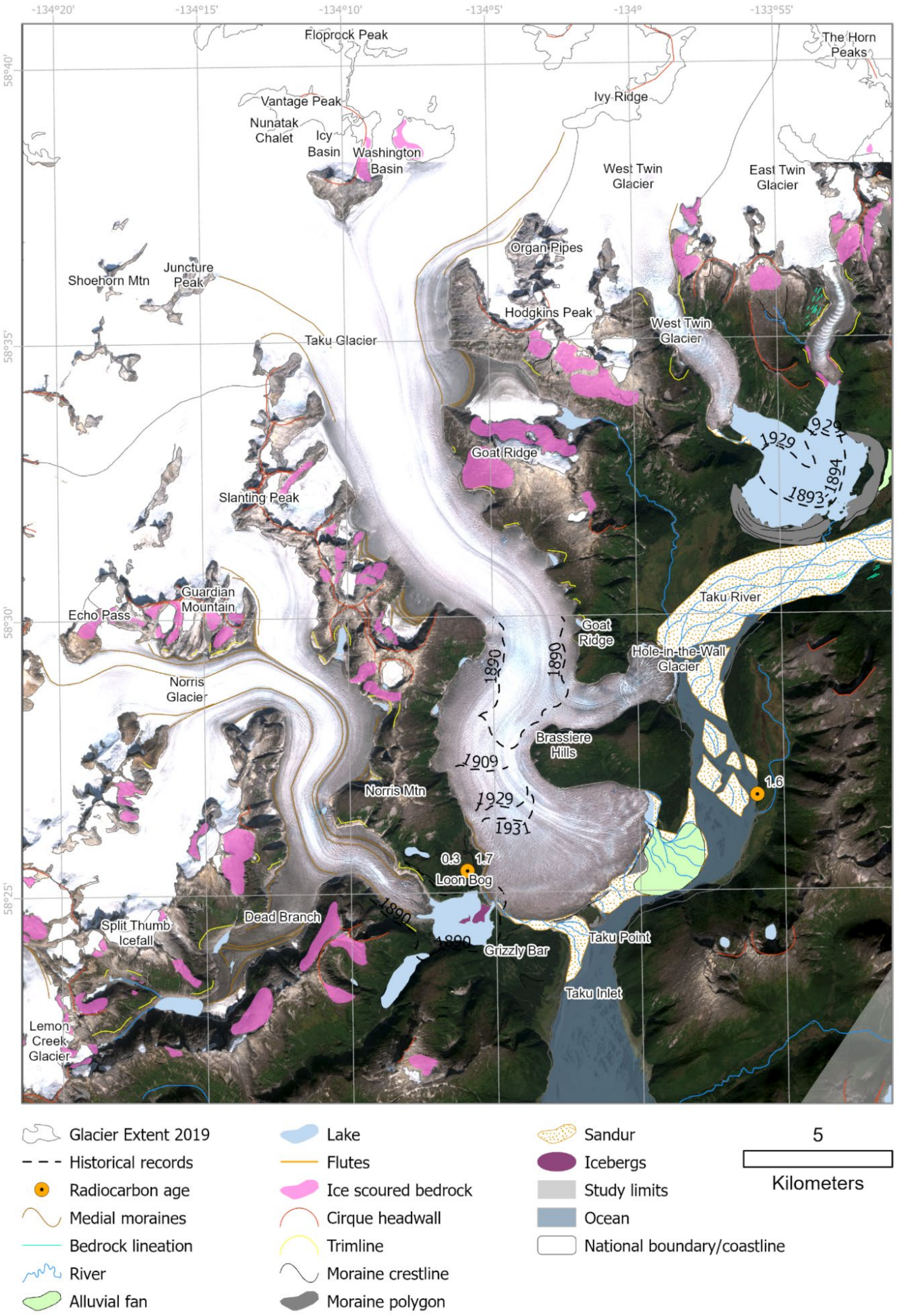

Supplementary figure 5. Taku Glacier, showing geomorphic, chronological and survey data used in the LIA reconstruction. Taku terminus was surveyed higher up-valley in 1890 AD by the United States Coast and Geodetic Survey and US Geological Survey Maps[17,28,29] (black dashed lines). Overlain on Sentinel-2A satellite image from 1st September 2019.

Herbert Glacier reached its LIA maximum, where it formed a substantial six-meter high moraine, in AD 1765[17] (Supplementary figure 1h). Significant recession (~2 miles) had occurred by 1948[17]. Eagle Glacier moraines were loosely dated to AD 1785-1787 by dendrochronology published in 1950[17], with recession of ca. 0.7 miles noted from this position in 1909-1910[20] and further recession of 0.7 miles by 1948 (*ibid*). Both these glaciers developed ice-marginal lakes during 20th century recession[16], leading to increased rates of recession due to calving. A similar history was noted at Mendenhall Glacier (near the town of Juneau), where the glacier in 1948 had receded from its LIA maximum (AD 1769) by two miles. Lemon Creek Glacier also formed LIA moraines dated to c. AD 1750 by dendrochronology[31]. These moraines demarcate the Holocene maximum extent of this glacier, 2.5 km downstream of the 1958 position.

In this work, we assume that the LIA maximum at Juneau Icefield occurred at AD 1770. However, due to the uncertainty in the timing of the LIA, and due to the presence of records in other locations indicating a later maximum[6,8,13,15], we also calculate rates of recession at from a maximum at 1880 AD and for an earlier LIA maximum at AD 1675[15,32].

4 SUPPLEMENTARY METHODS

4.1 Data sources, 1948 and 1979

Supplementary figures 6 and 7 below shows the source of the imagery used in the reconstruction of glacier area in 1948 and 1979. Aerial photographs were used wherever they were available. Where they were not available, such as over Canada, topographic maps for the main outlet glaciers from this era were used (Supplementary table 5). Some of the smaller glaciers peripheral to the icefield, especially in the northeast, were not viewable in the map or aerial photography, and so the earliest available imagery (in this case, the 1980s satellite imagery) was used. The date of the source imagery was recorded in all cases in attribute information and used when calculating rates of recession. The 1948 icefield reconstruction should therefore be considered a minimum, as a few glaciers were mapped using later imagery (Supplementary table 3), which is recorded in the attribute information. The date is taken into account when calculating annualised rates of change.

Aerial photograph imagery was more widely available in 1979, with Landsat 3 satellite data used for gap-filling where needed (Supplementary table 4). A list of all satellite imagery used is available in Supplementary table 6. The date of the source imagery is recorded in the attribute information of each glacier polygon, and this is accounted for (number of days) when calculating annualised rates of change.

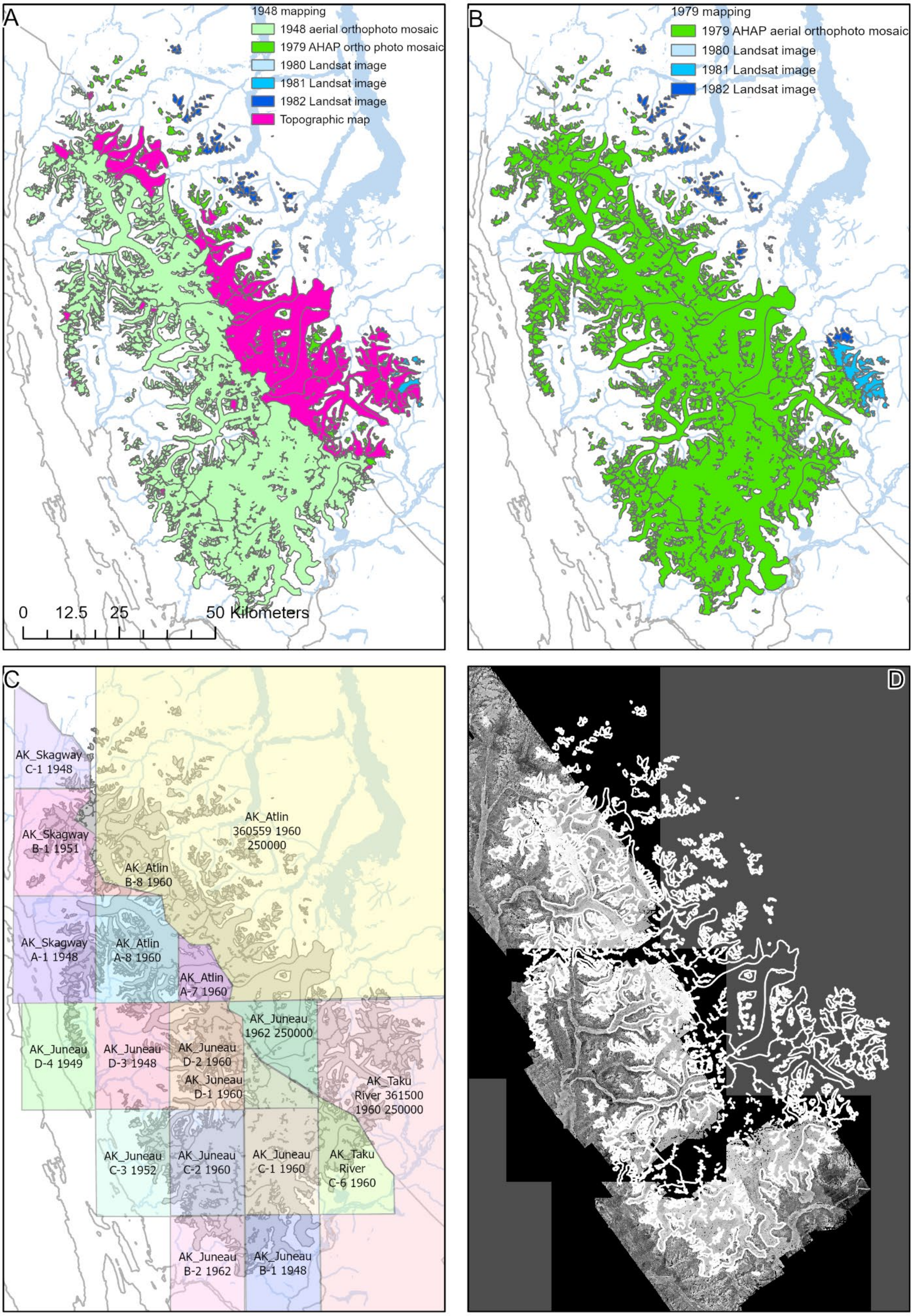

Supplementary figure 6. Sources of imagery used in A) 1948 and B) 1979 timeslices. Note that some data gaps in the USA imagery meant that holes were filled using the Topomaps. C) The different USGS Topomaps used. See Supplementary table 2 for more information. D) the 1948 ariel photograph mosaics over the USA. Aerial photographs are available from USGS Earth Explorer (see 'Data Availability'). New orthomosaics are available in Mendeley Data (see 'Data availability'). Historical

topographic maps are available from the USGS map viewer (https://ngmdb.usgs.gov/topoview/viewer/#4/52.19/-123.71) as georeferenced GEOTIFFS. AHAP: Alaska High Altitude Photography.**

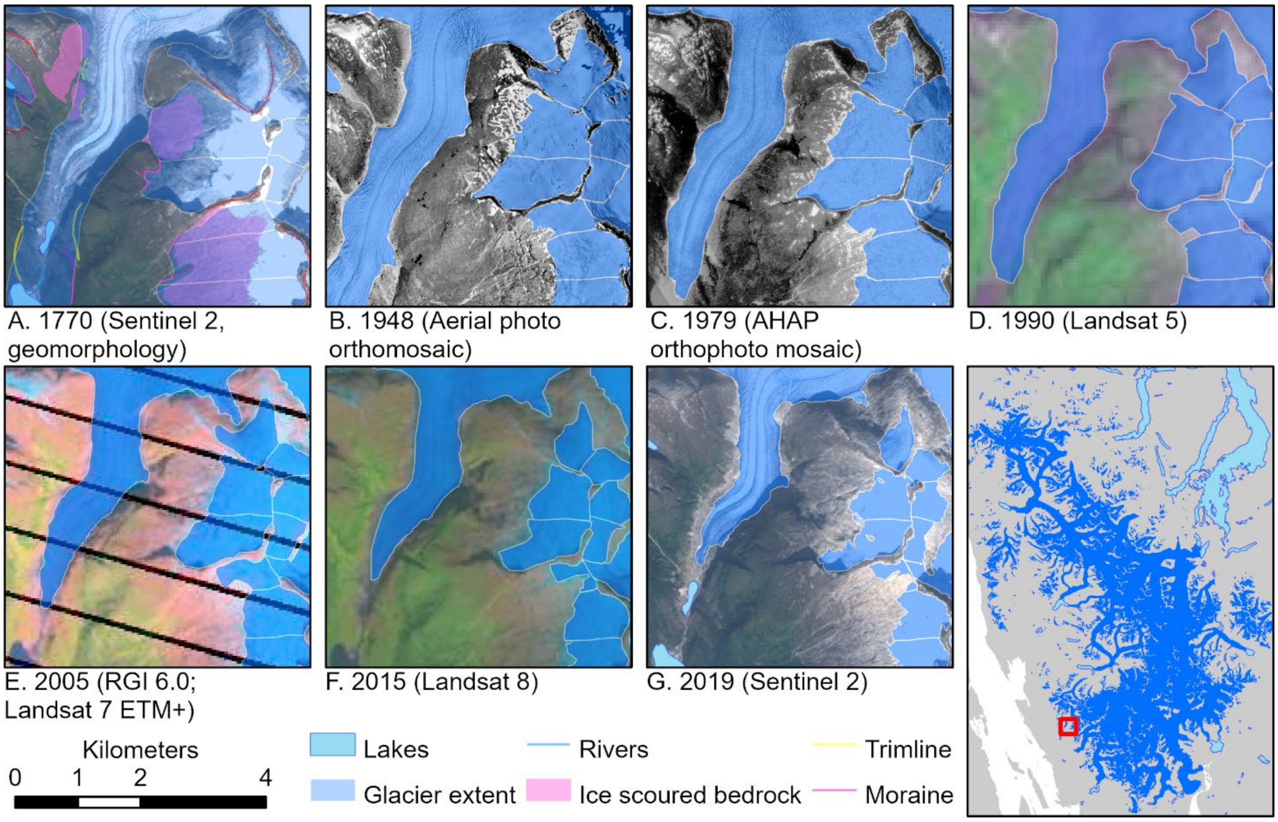

Supplementary figure 7. Imagery used in the reconstruction of glacier area in each timeslice. AHAP: Alaska High Altitude Photography.

The source imagery for each single glacier polygon is provided in the attribute information of the shapefile, and summarised below in Supplementary table 3.

Supplementary table 3. Source imagery for 1948 glacier outlines. AHAP: Alaska High Altitude Photography.

**Source imagery (1948 glacier outlines)**	**Number of glaciers**	**Area of glaciers (km^2)**	**% Glacier area**
1948 aerial orthophoto mosaic	658 (58.9%)	3169.9	66.6%
1979 AHAP orthophoto mosaic	203 (18.2%)	164.7	3.5%
1980 Landsat image	11 (0.98%)	3.9	0.08%
1981 Landsat image	18 (1.6%)	17.5	0.37%
1982 Landsat image	111 (9.9%)	79.6	1.67%
Topographic map	117 (10.5%)	1320.0	27.8%
Total	*1118*	*4755.5*	

Supplementary table 4. Source imagery for the 1979 glacier outlines. AHAP: Alaska High Altitude Photography.

**Source imagery (1979 glacier outlines)**	**Number of glaciers**	**Area of glaciers (km^2)**	**% Glacier area**
1979 AHAP orthophoto mosaic	956 (85.5%)	4395.1	95.9%
1980 Landsat image	11 (0.98%)	4.0	0.09%
1981 Landsat image	38 (3.4%)	92.2	2.0%
1982 Landsat image	113 (10.1%)	92.7	2.0%
Total	*1118*	*4584.0*	

4.2 Assessment of Topographic Maps

Reconstructing glacier extent used USGS topographic maps where aerial photographs were not available, or where there were gaps in the orthomosaics. Data was taken directly from information on the maps. Topographic maps were downloaded from the USGS map viewer (https://ngmdb.usgs.gov/topoview/viewer/#4/52.19/-123.71) as georeferenced GEOTIFFS. Our qualitative quality assessment (Supplementary table 5) used comments directly from the maps and compared topographic maps to the Arctic DEM, ASTER GDEM and ESRI Basemap (usually GeoEye). The location of topography (valleys, hills, lakes, rivers, etc) was compared in the datasets. Maps with a high degree of correspondence to the high resolution remotely sensed datasets were rated as 'high confidence'; highly stylised glaciers and a low degree of correspondence between lakes, rivers, glaciers and topography were rated as 'low confidence'. Maps where the aerial photographs were unclear were also rated as 'low confidence'. The date of the glacier extent was taken to be the latest date of the aerial photographs used. Unless otherwise specified, 1948 aerial photographs were presumed to have been taken on 13.08.1948.

Supplementary table 5. Assessment of USGS topographic maps used in 1948 icefield reconstruction. Map locations are shown in Supplementary Figure 6. Satellite imagery are available from USGS Earth Explorer (https://earthexplorer.usgs.gov/).

**Data from map sheets**							**Quality assessment**		
**Map ID**	**Area covered**	**Published / Revised**	**Aerial Photo**	**Annotated**	**Scale**	**Contour**	**Comments**	**Confidence level in map**	**Number of glaciers**
AK_Skagway A-1 359186 1948 63360	Western outlying glaciers. Tongass Range. Meade Glacier.	1951	August 1948		1:63,360	100 ft	Not field checked.	High	2
AK_Skagway B-1 359204 1951 63360	North-western Juneau Icefield. Tongass Range.	1951	July 1948		1:63,360	100 ft	Not field checked	High	5
AK_Skagway C-1 359220 1948 63360	North-western outlying glaciers. Alaska only.	1948	1948		1:63,360	100 ft	Not field checked. Small offset in map (135 m to the west) observed.	Medium	2
AK_Atlin_A-8 354103_1960_63360	Central Juneau Icefield. Meade Glacier. Mount London, Mount Service, Mount Poletica	1960	1948/1961	1960	1:63,360	100 ft	Not field checked. Not clear on aerial photograph coverage. Good topographic correspondence. Checked against 1948 topomap.	High	1
AK_Atlin_360559_1960_250000	British Columbia; eastern Juneau Icefield. Llewellyn Glacier.	1960	Unclear but Canadian portion copied from Atlin, 1954 and Skagway, 1957. Aerial photographs from 1948 used in map.		1:250,000	100 ft	Compiled in 1965 from USGS maps, surveyed 1960. Numerous glaciers not mapped (extent remains as 1980). Stylised glaciers. Lakes and highpoints show good correspondence. Offset is variable. Only lower tongues of glaciers in the valleys are adjusted as terminus change is clearly indicated.	Medium	28
AK_Juneau C-2 356344 1960 63360	Southwestern Juneau Icefield. West Branch Glacier.	1960	1948	1960	1:63,360	100 ft	Not field checked. Good correspondence to glaciers and topography.	High	3
AK_Juneau C-3 356345 1952 63360	Eagle Glacier.	1952	1948 and 1952		1:63,360	100 ft	Topography from aerial photographs by multiplex methods 1952. Aerial photographs taken 1948.	High	2

AK_Juneau D-1 356361 1960 63360	Alaska only. Upper parts of Matthes Glacier, Gilkey Glacier.	1960 / 1975	1948/1961	1960	1:63,360	100 ft	Not field checked. Not clear on aerial photograph coverage. Good correspondence	Low	3
AK_Juneau D-2 356362 1960 63360	Gilkey Glacier; Antler Glacier	1960	1948	1960	1:63,360	100 ft	Not field checked. Good correspondence but some offsets. Map contours do not match adjacent maps; height accuracies unclear.	Medium	7
AK_Juneau D-3 356367 1949 63360	Central western Juneau Icefield	1960 / 1963	1948	1948	1:63,360	100 ft	Not field checked. Good correspondence. Small offsets.	High	1
AK_Juneau D-4 356368 1949 63360	Western outlying glaciers	1953	1948		1:63,360	100 ft	Not field checked. Good correspondence	High	6
AK Taku River C-6 359507 1960 63360	Wright Glacier. Alaska only. West Twin / East Twin glaciers.	1960 / 1995	1948	1960	1:63,360	100 ft	Map not field checked.	High	3
AK_Taku River 361500 1960 250000	Southeastern Juneau Icefield; British Columbia portion used only	1960	Compiled in 1965 from USGS maps surveyed 1951 and 1960.		1:250,000	100 ft	Map not field checked. Rather generalised glaciers. Poor correlation to GeoEye imagery, Arctic DEM and ASTER GDEM.	Low	54

4.3 Source and date of satellite imagery, 1980s onwards

Supplementary table 6. Sources of satellite imagery used in this study

Sensor	Image ID	Path	Row	Date	Resolution	Swath
Landsat 3 MSS	LM30630181982225AAA03	63	18	13/08/1982	80 m	185 km
Landsat 3 MSS	LM20630181981149AAA04	63	18	29/05/1981	80 m	185 km
Landsat 3 MSS	LM20620191981220AAA03	62	19	08/08/1981	80 m	185 km
Landsat 3 MSS	LM30620191982260PAC00	62	19	17/09/1982	80 m	185 km
Landsat 3 MSS	LM30630191980218AAA03	63	19	05/08/1980	80 m	185 km
Landsat 3 MSS	LM30640181980219AAA03	64	18	06/08/1980	80 m	185 km
Landsat 4-5 MSS	LM50580191992163PAC04	58	19	11/06/1992	30 m	183 km
Landsat 4-5 MSS	LM50580191990269PAC00	58	19	26/09/1990	30 m	183 km
Landsat 4-5 MSS	LM50580191990237PAC00	58	19	25/08/1990	30 m	183 km
Landsat 4-5 MSS	LM50580191990205PAC00	58	19	24/07/1990	30 m	183 km
Landsat 4-5 MSS	LM50570201992140PAC00	57	19	19/05/1992	30 m	183 km
Landsat 4-5 MSS	LM50570191990230PAC00	57	19	18/08/1990	30 m	183 km
Landsat 4-5 MSS	LM50570191990198PAC00	57	19	17/07/1990	30 m	183 km
Landsat 4-5 MSS	LT50580181991176PAC00	58	18	25/06/1991	30 m	183 km
Landsat 7 ETM+ SLC-off (1999-2003)	LE07_L1TP_058018_20050810_20160924_01_T1.tif	58	18	10/08/2005	30 m	185 km
Landsat 7 ETM+ SLC-off (1999-2003)	LE07_L1TP_058019_20050810_20160924_01_T1.tif	58	19	10/08/2005	30 m	185 km
Landsat 7 ETM+ SLC-off (1999-2003)	LE07_L1TP_056019_20050812_20160925_01_T1.tif	56	19	12/08/2005	30 m	185 km
Landsat 8 OLI/TIRS	LC08_L1TP_057019_20140820_20170304_01_T1	57	19	20/08/2014	30 m	185 km
Landsat 8 OLI/TIRS	LC08_L1TP_057019_20150706_20170226_01_T1	57	19	06/07/2015	30 m	185 km
Landsat 8 OLI/TIRS	LC08_L1TP_058019_20140827_20170303_01_T1	58	19	27/08/2014	30 m	185 km
Landsat 8 OLI/TIRS	LC08_L1TP_058019_20150814_20170226_01_T1	58	19	14/08/2015	30 m	185 km
Landsat 8 OLI/TIRS	LC08_L1TP_059018_20140802_20170304_01_T1	59	18	02/08/2014	30 m	185 km
Landsat 8 OLI/TIRS	LC80580192014239LGN00	58	19	27/08/2014	30 m	185 km
Sentinel-2B	L1C_T08VML_A012967_ 20190830T202101			30/08/2019	10 m	290 km
Sentinel-2B	L1C_T08VNM_A013067_ 20190906T200951			06/09/2019	10 m	290 km
Sentinel-2A	L1C_T08VNL_A021904_ 20190901T201750			01/09/2019	10 m	290 km
Sentinel-2A	L1C_T08VNK_A021904_ 20190901T201750			01/09/2019	10 m	290 km
Sentinel-2A	L1C_T08VMM_A021990_ 20190907T203043			07/09/2019	10 m	290 km

4.4 Analysis of uncertainty in glacier area

Methods of deriving an uncertainty estimate of glacier area are explained in detail in Methods, and are illustrated below in Supplementary Figure 8 and in Supplementary Table 7.

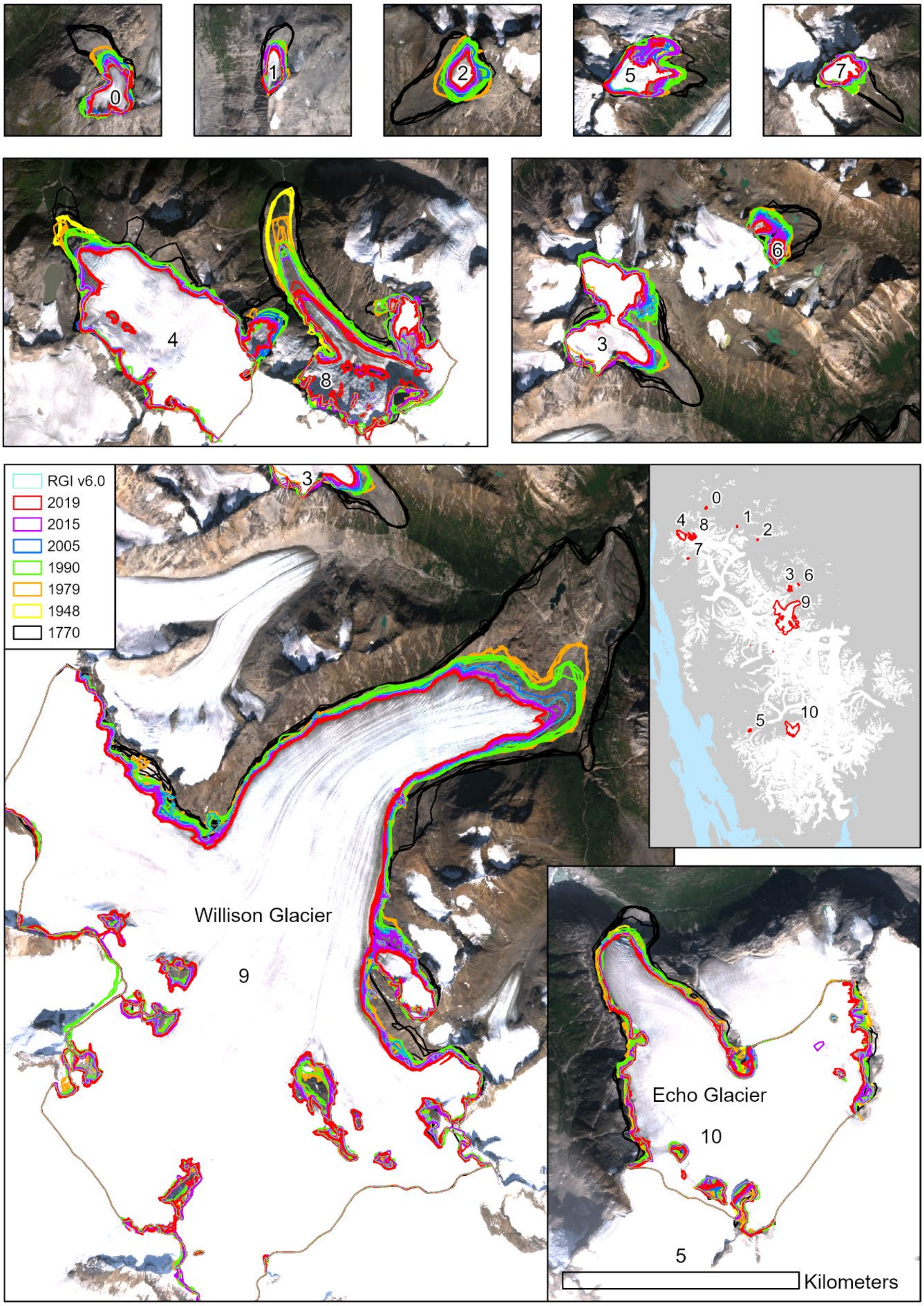

Supplementary figure 8. Analysis of uncertainty in glacier area mapping. Scale consistent in all glaciers. Overlain on Sentinel 2A imagery (cf. Supplementary table 6)

Supplementary table 7. Analysis of uncertainty in glacier area mapping. 11 glaciers were independently mapped 7 times. Std Dev: Standard Deviation. CI: Confidence Interval. RSQ: R squared value. RSD: Relative Standard Deviation.

Year	Image resolution	Mean Std Dev	Mean % Std Dev (RSD)	Mean 95% CI	RSQ (Mean area, CI)	p-value (mean area, CI)
LIA	Geomorphology (10 m)	0.47	5.86	0.35	0.88	0.00
1948	4 m	0.04	0.63	0.03	0.84	0.03
1979	4 m	0.17	4.09	0.13	0.76	0.00
1990	30 m	0.09	3.51	0.07	0.85	0.00
2005	30 m	0.09	3.02	0.06	0.80	0.00
2015	30 m	0.12	5.87	0.09	0.88	0.00
2019	10 m	0.03	1.27	0.02	0.84	0.00

Supplementary table 8. Total summed area of all glaciers with no DEM coverage in volume change estimates, LIA-2020. LIA: "Little Ice Age". DEM: Digital Elevation Model.

	LIA-1948	1948-1979	1979-2000	2000-2010	2010-2020
**% area no DEM coverage**	44.6%	16.8%	3.8%	0%	0%
**Area no DEM coverage (km^2)**	2614.8 km^2	798.2 km^2	163.0 km^2	0	0

5 SUPPLEMENTARY RESULTS

5.1 Little Ice Age glaciation

Glacial geomorphology at the LIA is presented in Ref. [23]. This was ground-truthed and checked during fieldwork to Juneau Icefield (focused on Taku Glacier, Echo Glacier, Herbert Glacier, Twin Glacier Lake, Mendenhall Glacier) in July 2022, and is now supplemented by additional geomorphological mapping of 5 alluvial fans around Taku Glacier (see Supplementary figure 9 below), and 12 trimlines and 64 moraines around some terminal glaciers (Herbert, Mendenhall, Twin Glaciers). The landform inventory is available from Ref.[23] or from the authors by request. See also Figures 3 and 4 (main manuscript).

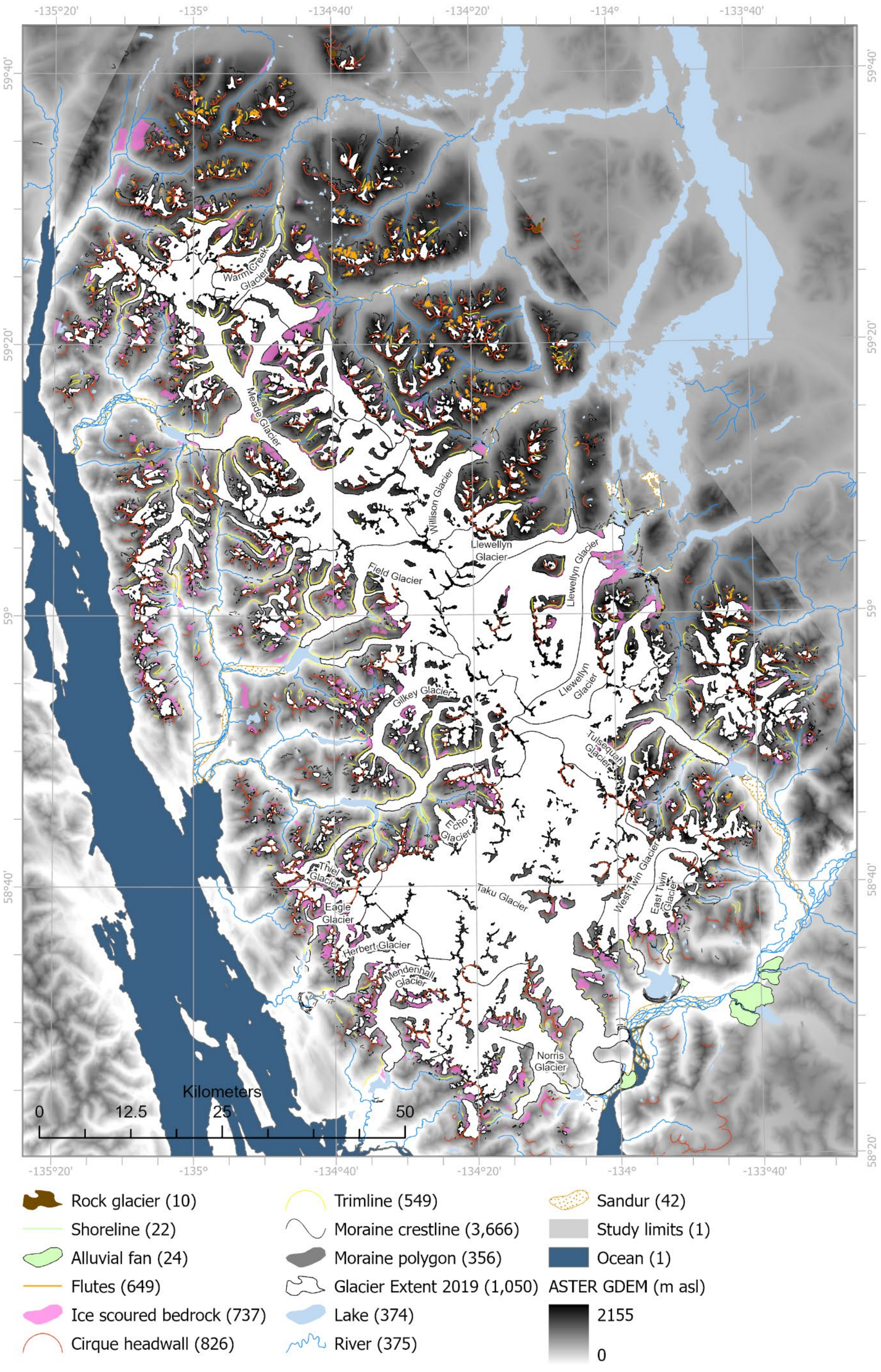

Supplementary figure 9. Geomorphological map and landform inventory for Juneau Icefield. Overlain on ASTER GDEM, the Global Digital Elevation Model produced by ASTER, courtesy of NASA/JPL-Caltech.

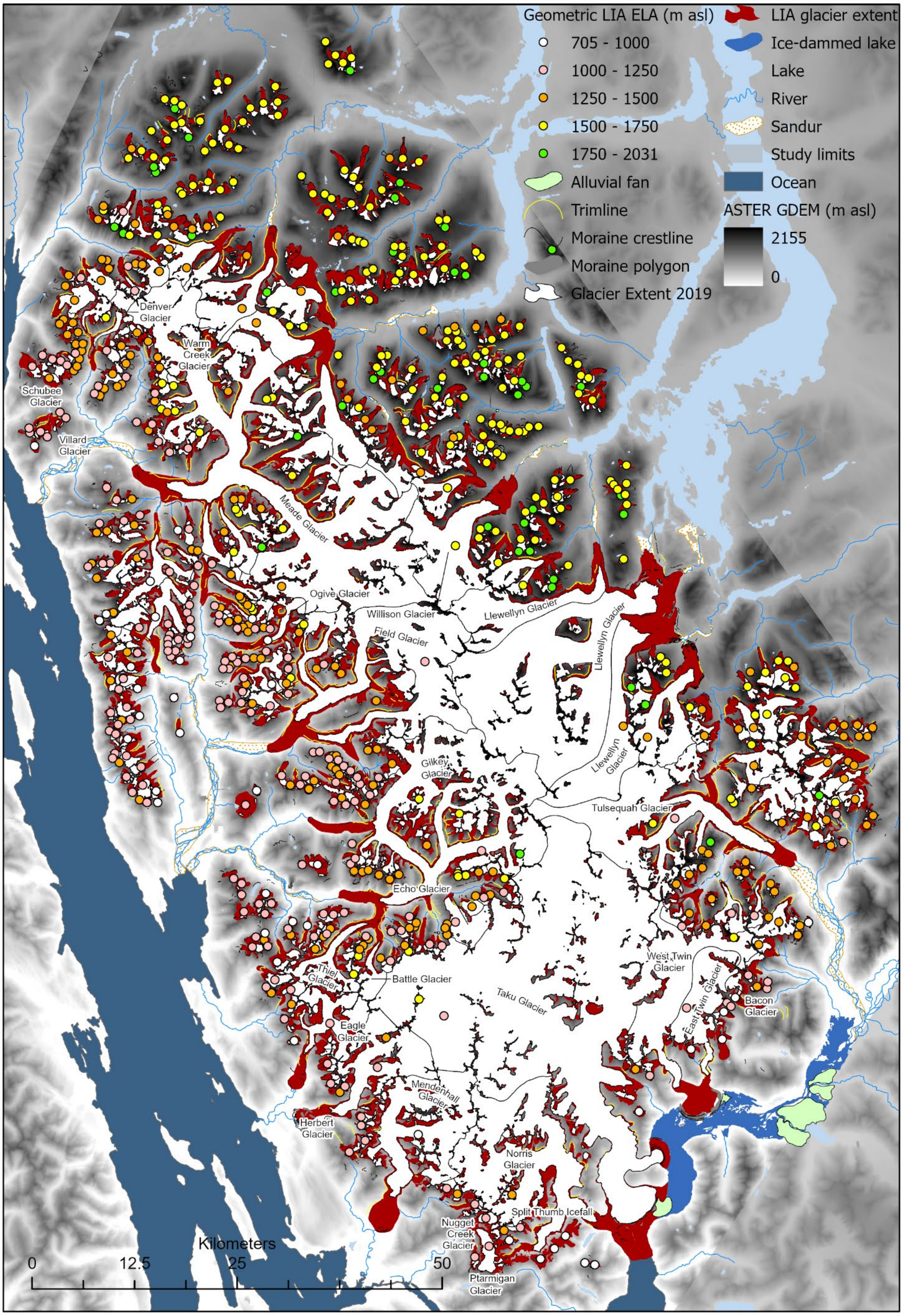

Supplementary figure 10. Reconstruction at the LIA. Circles show height of the reconstructed geometric ELA_{LIA} (see main legend). LIA: "Little Ice Age". ELA: Equilibrium Line Altitude. Overlain on ASTER GDEM, the Global Digital Elevation Model produced by ASTER, courtesy of NASA/JPL-Caltech.

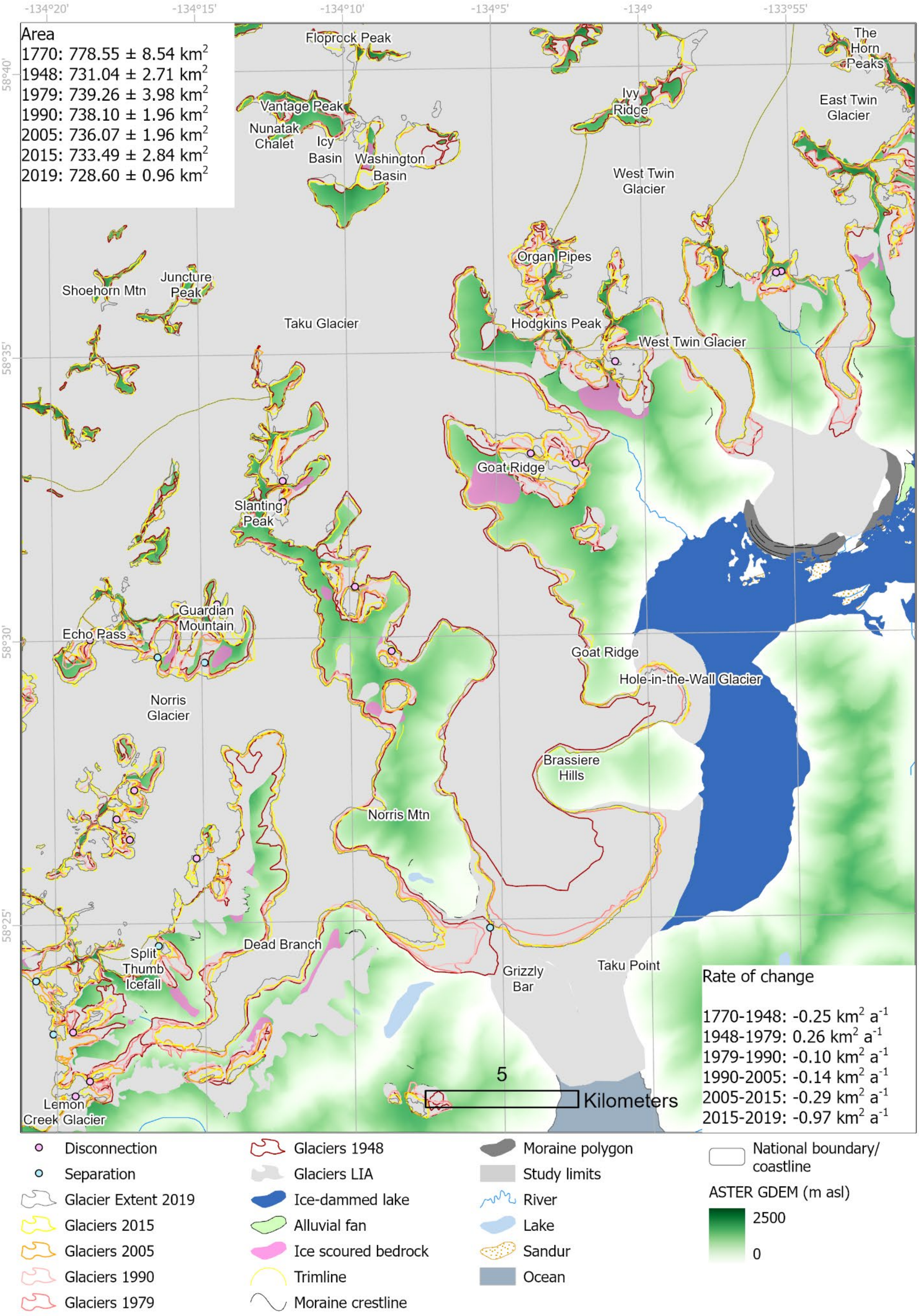

Supplementary figure 11. Reconstructed glacier area change for Taku Glacier. LIA: "Little Ice Age". Overlain on ASTER GDEM, the Global Digital Elevation Model produced by ASTER, courtesy of NASA/JPL-Caltech.

Supplementary table 9. Examples of outlet glacier length and area change between the LIA and 2019 AD. LIA: "Little Ice Age".

Glacier name	Terminus recession (m), LIA-2019	Area change, LIA-2019 (km^2)	Terminal environment change
Tulsequah	2951	19.40	Growth of a small proglacial lake, 4.4 km^2 and 2821 m long
Meade	4616	69.31	Growth of a large proglacial lake 3660 m long filled with icebergs, indicating present-day mass loss through calving.
Gilkey	4940	55.46	Development of a large proglacial lake (5.65 km^2) and separation of tributary glacier termini as they recede up adjoining valleys. Terminus calves icebergs into the proglacial lake.
Field	5134	44.09	Terminus separation, forming two distinct tongues in different valleys and a large proglacial lake filled with icebergs, suggesting substantial calving.
Eagle	4372	15.23	Recession of a piedmont lobe glacier into a confined and steepening valley. Growth of some small lakes in the over-deepening behind the LIA moraines.
			Recession of 1001 m from ~1770-1908 and 1021.9 m from 1908-1948.
Mendenhall	4285	31.12	Recession of a piedmont lobe glacier and development of a large proglacial lake. The glacier is now receding out of the lake, with reduction in calving.
			Recession of 787 m from ~1770-1909, 715 m from 1909-1931, and 935 m from 1931-1948.
Herbert	4770	10.88	Recession of a piedmont lobe glacier into the confining narrow valley. Recession of 800 m from LIA to 1909, 1550 m from 1909-1948, and 763 m from 1948-1979.
Norris	6348	43.237	Recession from a calving margin, confluent with Taku glacier, at Taku Inlet. Formation of an ice-dammed lake up-valley of the terminus.
West Twin Glacier	3198	16.80	Recession of a piedmont lobe glacier from the wide valley floor and into the confining valley. Development of a large proglacial lake in the forefield, which the glacier is now receding out of, with cessation of calving.
Meade	4785	68.92	Recession of the terminus and separation of several tributary glaciers into adjoining valleys. Formation of a proglacial lake currently filled with icebergs, 4.7 km^2 in area.
Llewellwyn	4821	32.60	Recession of broad piedmont lobe glacier up-valley, with formation of proglacial lake in the over deepening behind the moraines.

Taku	LIA to 1890: 11,420 m recession<br>1890-1948: 4964 m advance	Taku Glacier receded by over 11 km from the LIA to the 1890 surveyed position[17,28]. The 1948 terminus is 3130 m behind the LIA extent (Supplementary figure 11). Taku Glacier calved directly into the wide Taku River at this time. It then advanced again to 2005, whereupon the terminus remained fairly stable. Losses in other parts of the glacier resulted in an overall shrinkage, however.

5.2 Glacier change, LIA to 1948

In total, 91.04% of glaciers receded from AD 1770-1948 AD, though this is a minimum estimate as it was not possible to accurately reconstruct LIA glacier extent for all glaciers. Sparse historical records[17] suggest relatively slow but consistent recession from the LIA to ~1900, (Supplementary table 9), with accelerating recession from the early 1900s to 1948 and then onwards into the late twentieth century.

Supplementary table 10. Rates of glacier area change for each time slice, for different kinds of glacier. "Advancing" is the number of glaciers with increased glacier area.

	GLACIERET						MOUNTAIN GLACIER					
YEAR	AREA (km^2)	n	ΔA % a^{-1}	ΔA km^2 a^{-1}	# Advancing	Mean ΔA % a^{-1}	AREA (km^2)	n	ΔA % a^{-1}	ΔA km^2 a^{-1}	# Advancing	Mean ΔA % a^{-1}
1770	165.12 ± 20.82	304					797.39 ± 47.98	667				
1948	84.65 ± 2.54	297	-0.27	-0.01	17	0.25	522.14 ± 6.69	635	-0.19	-1.55	36	0.21
1979	83.55 ± 19.86	296	-0.05	-0.01	102	-0.39	498.35 ± 44.30	635	-0.14	-0.73	220	0.01
1990	79.32 ± 11.86	295	-0.55	-0.07	121	0.24	486.49 ± 26.35	635	-0.25	-1.23	236	0.11
2005	62.12 ± 9.23	290	-1.47	-0.17	30	1.41	411.45 ± 20.66	631	-1.07	-5.20	58	1.15
2015	42.68 ± 14.43	277	-3.32	-0.70	2	3.38	339.61 ± 32.09	626	-1.82	-7.47	8	2.42
2019	26.82 ± 2.92	304	-7.76	-0.36	0	8.84	279.60 ± 6.48	588	-3.73	-12.68	2	5.56
	VALLEY GLACIER						OUTLET GLACIER					
YEAR	AREA (km^2)	n	ΔA % a^{-1}	ΔA km^2 a^{-1}	# Advancing	Mean ΔA % a^{-1}	AREA (km^2)	n	ΔA % a^{-1}	ΔA km^2 a^{-1}	# Advancing	Mean ΔA % a^{-1}
1770	975.43 ± 14.80	147					3477.01 ± 28.16	40				
1948	839.19 ± 4.21	147	-0.08	-0.77	11	0.11	3309.53 ± 12.55	40	-0.03	-0.94	3	0.05
1979	781.07 ± 13.78	147	-0.22	-1.85	15	0.28	3220.98 ± 19.70	40	-0.09	-2.85	5	0.15
1990	753.57 ± 7.77	147	-0.34	-2.66	21	0.43	3176.71 ± 9.84	40	-0.12	-3.98	9	0.05
2005	683.47 ± 6.69	147	-0.63	-4.78	4	0.82	3081.62 ± 11.09	40	-0.20	-6.39	3	0.52
2015	620.74 ± 9.54	146	-0.98	-6.66	1	1.47	2998.44 ± 13.36	40	-0.30	-9.16	0	0.80
2019	570.86 ± 2.25	145	-1.69	-10.47	1	2.49	2939.05 ± 4.24	40	-0.40	-12.02	2	1.69

Supplementary table 11. Rates of glacier area shrinkage (% a^{-1}) for specific Juneau Icefield glaciers. RGI ID: the ID of the glacier in the Randolph Glacier Inventory v6.0.

Glacier name	RGI ID	1770-1948	1948-1979	1979-1990	1990-2005	2005-2015	2015-2019
**GILKEY**	RGI60-01.00704	0.01	0.14	0.18	0.47	0.60	0.14
**FIELD**	RGI60-01.01520	0.01	0.16	0.23	0.35	0.67	0.14
**EAST TWIN**	RGI60-01.23663	0.06	0.06	0.25	0.11	0.16	0.85
**WEST TWIN**	RGI60-01.01514	0.06	0.05	0.11	0.06	0.13	0.38
**MENDENHALL**	RGI60-01.00709	0.09	0.11	0.17	0.33	0.45	0.52
**MEADE**	RGI60-01.01524	0.01	0.07	0.18	0.11	0.39	0.61
**LLEWELLWYN**	RGI60-01.27103	0.00	0.09	0.11	0.24	0.03	0.42
**TAKU**	RGI60-01.01390	0.07	-0.04	0.01	0.02	0.04	0.13
**TULSEQUAH**	RGI60-01.01521	0.00	0.05	0.11	0.35	0.26	0.36
**THIEL**	RGI60-01.01242	0.00	0.36	0.87	0.77	1.31	0.77
**HERBERT**	RGI60-01.01245	0.02	0.12	0.06	0.28	0.39	-0.06
**DENVER**	RGI60-01.01403	0.04	0.35	0.02	0.74	0.58	0.86
**LEMON CREEK**	RGI60-01.01104	0.07	0.37	0.26	0.35	1.70	0.75

Often variable rates of recession are caused by terminus dynamics as glaciers interact with the large proglacial lakes forming in front of their termini. For example, West Twin Glacier experienced fast rates of glacier recession in the early 1900s as its terminus receded across the proglacial lake (Supplementary tables 9, 11, 12). These rates slowed as the glacier reached a natural stabilising pinning point at the narrow mouth of the valley, before again accelerating.

Supplementary table 12. Record of annual average rates of glacier terminus change (length; m a^{-1}) for glaciers where older historical records are available.

Glacier	~1770-1890	1890-1929	1929-1948	~1770-1908	1908-1948	1948-1979	1979-1990	1990-2005	2005-2015	2015-2019
Eagle				-7.25	-25.6	-26.4	-27.42	-30.5	-50.1	-66.3
Herbert				-5.70	-39.7	-24.6	-21.0	-23.6	-44.4	-30.2
Mendenhall				-5.66	-40.2	-23.0	-44.3	-53.7	-50.5	-48.0
West Twin Glacier	-9.7	-38.13	-147.7			-16.9	-20.6	-18.9	-14.3	-15.0
Taku	-69.3	+72.8	+122.4			+47.2	+8.5	+6.7	+12.9	-14.2

Across Juneau Icefield and its peripheral glaciers, slow volume loss is observed from AD 1770 – 1948 AD, at -0.65 ± 0.92 $km^3 a^{-1}$ (Figure 6e, f). These rates are correspondingly lower (-0.39 ± 0.55 $km^3 a^{-1}$) for an earlier (AD 1675) timing of the LIA. They are higher (-1.70 ± 2.40 $km^3 a^{-1}$) for a later (AD 1880) timing of the LIA. Calculated uncertainties are high because the LIA DEM only covers glacier ablation areas and the 1948 DEM is interpolated over some of the eastern portion of the icefield.

Short periods of faster rates of volume loss or readvances cannot be excluded due to a paucity of data prior to 1948, and Taku Glacier in particular is known to have receded behind the 1948 extent by 1890 AD (Supplementary figure 11, Supplementary table 12), followed by a readvance[17]. However, this behaviour is likely to be anomalous, and related to Taku Glacier's evolving terminal environment[26,33]. Temperature records from ice cores and tree rings[6,9] suggest uninterrupted, steady warming between the LIA and 1948, which would likely result in steady glacier recession, though temperatures reconstructed from varved lake

sediments north of the study area suggest do some cooling from 1870-1900 (Figure 6a)[11]. Further, historical records from 1890 and 1908-1910 AD from Twin Glacier Lake (Supplementary table 12), Mendenhall (Figure 2), Eagle and Herbert Glacier (Figure 5) all indicate continued recession from the LIA to 1948 AD[17]. There are few moraines between the mapped LIA neoglacial maximum and the 1948 extent (Supplementary figure 9), which would suggest a steady recession, uninterrupted by readvances.

5.3 Glacier change, 1948 - 1979

Glaciers remained stable from 1948-1979 AD, receding overall at 5.48 km^2 a^{-1} (0.12 % a^{-1}). During this period, there were stable air temperatures (with a cooler decade from 1965-1975) and increasing winter precipitation[9,34] (Figure6c, h, j; Supplementary figure 3, 4; Supplementary table 2). 342 glaciers (30.6%) advanced from 1948-1979, though due to poor aerial photograph coverage in the Canadian part of the icefield in 1948 (Supplementary figure 6), this is a minimum estimate.

Icefield-wide volume loss from 1949 to 1979 AD was -1.06 ± 0.74 km^3 a^{-1} (Figure 6d). Thinning is concentrated in the glacier tongues, below the ELA, with elevation change on the icefield plateau close to zero (Figure 7). On Field Glacier, glacier thinning reaches an elevation of 1200 m asl, and 1246 m on Ogive Glacier. Thinning greater than 5 m is observed on Gilkey Glacier up to 1520 m asl, Meade Glacier to 1230 m asl, on Eagle Glacier to 1230 m asl, and on Herbert Glacier to 980 m asl. Rates of thinning (mean dh/dt across the glacier) were low (mean -0.27 m a^{-1}, Figure 6e), with the highest values from Ogive Glacier (area-averaged elevation change of -1.58 m a^{-1}) and nearby valley glaciers. Other rapidly thinning glaciers include Thiel (-1.09 ma^{-1}) and Denver (-0.85 m a^{-1}). Glacier area-averaged elevation change on Taku Glacier was very slightly positive in this time period. Taku Glacier terminus thickened and advanced 1,486 m between 1948 and 1979 as it began to fill in the forefield over-deepening with sediment[33], with a total area growth of 8.22 km^2. Shoaling sediment is visible in front of the terminus in the 1979 aerial photographs.

5.4 Glacier change, 1979 – 2005

Rates of glacier recession increased after 1979, reaching 8.33 km^2 a^{-1} (0.18 % a^{-1}, 1979-1990) and 17.54 km^2 a^{-1} (0.39 % a^{-1}, 1990-2005). 387 glaciers (34.6%) advanced from 1979-1990. Advances were restricted to small Glacierets and Mountain Glaciers, and likely reflects increased snowfall at this time (Figure 4f) and the faster response time of the smaller glaciers. From 1990-2005, 95 glaciers (8.5%) advanced. However, the results from the 1948-1979 and 1979-1990 time periods indicate no statistically significant change in rate of recession (% a^{-1}) ($t(751)$, p = 0.96). Rates of recession (% a^{-1}) were statistically faster from 1990-2005 (M=1.16, SD=1.20) than from 1979-1990 (M=0.18, SD=1.37), ($t(1114)$, p < .001).

Icefield-wide volume loss from 1979-2000 reached -3.72 ± 1.57 km^3 a^{-1}, indicating an acceleration of thinning compared with the 1948-1979 time period. Median rates of area-averaged elevation change (dh/dt) for outlet glaciers reached -0.48 m a^{-1}, with valley glaciers at -0.72 m a^{-1} (Figure 6f, g). Thiel and Ogive were again the fastest-thinning glaciers in 1979-2000 (glacier-wide mean thinning of -2.43 and -2.03 m a^{-1} respectively). The terminus of Taku Glacier thickened by 20 m over this time period (1979-2000), though elevation change on the plateau was not observable. Taku Glacier advanced between 1979 and 1990 (a distance of just 140 m) (Supplementary tables 9, 12). Terminus thickening of up to 70 m allowed the Hole-In-The-Wall outlet to flow over a low col by the 1940s, forming a large piedmont glacier on the lowlands by 1960. Thinning was observed on the remainder of outlet glacier tongues across the rest of the icefield; the terminus of Meade and Field glaciers each thinned by up to 100 m. Gilkey Glacier thinned by up to 80 m, and similar values were observed on Mendenhall (Figure 7).

5.5 Glacier change, 2005-2019

From 2015-2019, 99.52% of glaciers receded, and 100% of glaciers mapped in 2019 had receded when compared with the 2005 inventory from the RGI[35]. When compared with the 2005 inventory, 10.0% of the icefield had disappeared by 2019 AD. Glacier recession accelerates after 2005, reaching total areal losses of 25.36 km^2 a^{-1} (0.60 % a^{-1}, 2005-2015) and then 38.47 km^2 a^{-1} (0.96 % a^{-1}, 2015-2019) (Figure 6a). Substantial areal losses were from the main Icefield's outlet glaciers, which shrank at a total summed rate of 12.02 km^2 a^{-1} from 2015-2019, a large increase in the rate observed from 2005-2015 (9.16 km^2 a^{-1}) (Supplementary table 10). Icefield-wide, this indicates a seven-fold increase in recession from 2015-2019 relative to 1948-1979, and a fivefold increase relative to 1979-1990 (Figures 5, 6a, b, c, g). The normalised total summed rate of glacier change (% a^{-1}) increased sharply after 2005 (Figure 6b, c; Table 1;Supplementary table 10) and was 1.5 times as fast from 2015-2019 compared with 2005-2015 (Table 1). In all measures, the fastest rates of recession were reached in 2015-2019 (Figure 6). When compared with rates from 1948-2005, this indicates an exponential acceleration of glacier recession across the icefield (Figure 6b, c, g).

As a proportion of their area, the smallest glaciers shrank fastest from 2015-2019 (Figure 6d; Supplementary figure 12). Mountain glaciers shrank at total summed rate of 3.73 % a^{-1} from 2015-2019, and glacierets shrank at 7.76 % a^{-1}, whilst outlet glaciers shrank at just 0.40 % a^{-1} (Figure 6b; Supplementary figure 12). Supplementary figure 12 shows how, from 2015-2019 AD, glaciers peripheral to the main icefield shrank fastest relative to their area, whilst the main outlet glaciers had the lowest normalised rates of recession. A comparison of glaciers east and west of the ice divide and lacustrine versus land-terminating glaciers found no statistically significant difference in recession. Glaciers that have a significant proportion of debris (>10% debris cover by area) are receding more slowly; debris-rich glaciers are receding at a mean rate of 5.51 % a^{-1} compared with 3.91 % a^{-1} for debris-free glaciers ($t(982) = 2.4$, $p = 0.00$). However, glaciers with >10% debris cover are thinning faster (mean of -1.19 m a^{-1} versus 0.76 m a^{-1} for debris-free glaciers) ($t(982) = 5.76$, $p = 0.00$). This supports downwasting rather than areal recession for those glaciers with the most debris.

Icefield-wide volume loss from 2000-2010 AD was 30.79 ± 10.13 km^3, and doubled to 59.09 ± 7.99 km^3 from 2010-2010 AD. This means that the icefield lost 5% of its volume in the period from 2010-2020 alone. Rates of volume loss are rapidly accelerating (Figure 6d, e, Table 2), with outlet glaciers exhibiting the fastest rates of thinning (dh/dt in Figure 6e), closely followed by valley glaciers. Thinning occurred across the plateau (Figure 7). Thinning at several outlet glacier termini is over 5 m a^{-1} (including Eagle, Gilkey, Field glaciers). Decreased thinning right at the snout of some glaciers, such as Tulsequah, indicates that these outlet glaciers are likely floating at their terminus, with thinning from below rather than the ice surface (Figure 7).

Taku Glacier has lost marginal glacier area since 1979, resulting in a slight recession (Supplementary table 11). Importantly, glacier area change is within or very close to mapping uncertainties from 1979 to 2015. From 2015-2019, the Taku Glacier area loss of 4.88 km^2 exceeds the mapping uncertainty of 0.96 km^2. Although terminus retreat is marginal at the snout from 2015-2019 (Supplementary table 11), there is ice loss in other parts around the margin of the glacier. The areal recession of Taku Glacier is now accelerating, rising from 0.29 km^2 a^{-1} (0.04 % a^{-1}, 2005-2015) to 0.97 km^2 a^{-1} (0.13 % a^{-1}, 2015-2019 AD) (Supplementary figure 11).

Mendenhall Glacier has receded across a large lake across the last 70 years, with substantial losses from the terminus as a result of increased calving due to terminus flotation [36,37]. The terminus is now partly grounded above lake-level, with a greatly reduced calving, no flotation of the terminus, and reduced frontal

ablation potential from a much narrower lacustrine terminus. This has resulted in a slight slowing of the rate of terminus recession (Supplementary table 12). The rate of terminus recession at Herbert Glacier has also slowed in recent years, likely also due to recession into the narrow valley and away from the proglacial lake.

In contrast, Gilkey Glacier receded fastest from 2005-2015 (0.60 % a^{-1}), with rates of recession slowing from 2015-2019. Field Glacier receded at 0.67 % a^{-1} from 2005-2015 and then recession also slowed, at 0.14 % a^{-1} from 2015-2019. Significant thinning (7 m a^{-1}) is now occurring 1.1 km up from the glacier terminus (Figure 7), though the area at the terminus, where rifting and calving occurs, is not thinning, due to ice flotation in the proglacial lake. Gilkey and Field glaciers both receded back across an over-deepened forefield filled with a proglacial lake. In the 2019 imagery, abundant calving is visible in these lakes, again indicating substantial frontal ablation (cf.[23]). A slowdown of recession for these glaciers may therefore indicate stretching and flotation of the ice as it thins over an over-deepened basin.

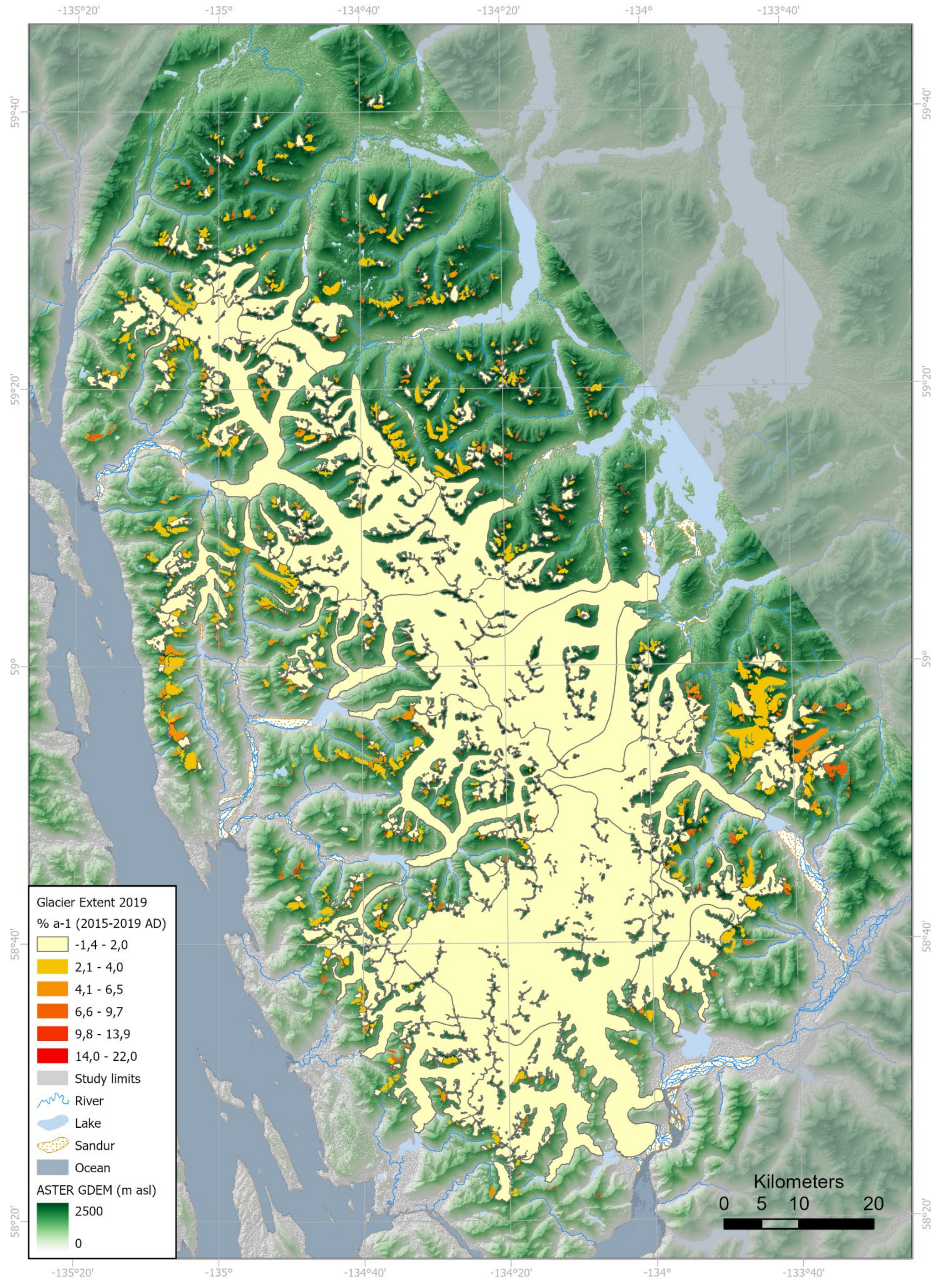

Supplementary figure 12. Glacier area change (% a[-1]) for 2015 to 2019 AD. Overlain on ASTER GDEM, the Global Digital Elevation Model produced by ASTER, courtesy of NASA/JPL-Caltech.

5.6 Glacier snowlines

Glacier snowline data were manually mapped from end-of-summer cloud-free Sentinel 2 satellite imagery (black and white, band 4 images) annually from 2019-2023 AD (Supplementary figure 13; Supplementary table 13). For each glacier, the mean elevation of multiple snowlines from one image is calculated. Mean end of summer snowline elevations for key named glaciers are shown in Supplementary table 14 and the full dataset is provided in the Source Data and in the shapefile of glacier snowlines in the Supplementary Data. The location of snowlines with respect to glacier outlines and the plateau is shown in Supplementary figure 13.

Supplementary table 13. Summary dataset of icefield-wide snowline elevation for end of summer snowlines from 2019 to 2023.

Year	Average elevation (m)			Average slope	No. Observations
	Minimum snowline	Maximum snowline	Mean snowline		
2019	1575.7	1652.7	1611.9	17.3	178
2020	1403.5	1492.2	1444.3	17.1	178
2021	1383.0	1450.5	1415.8	15.1	168
2022	1487.9	1553.0	1518.3	16.8	253
2023	1473.7	1532.9	1501.7	16.5	245

Supplementary table 14. Icefield-wide snowline data for named key glaciers from 2019 to 2023 AD. RGI ID: the ID of the glacier in the Randolph Glacier Inventory v6.0.

		Mean snowline elevation (m)				
**Glacier Name**	**RGI ID**	**2019**	**2020**	**2021**	**2022**	**2023**
Bacon Glacier	RGI60-01.01150	1369.8		1279.9	1105.3	
Battle Glacier	RGI60-01.00686	1539.8	1212.0	1461.7	1475.1	
Denver Glacier	RGI60-01.01403	1671.2	1119.4	1088.7		1636.9
Eagle Glacier	RGI60-01.01519	1430.5	1257.9	1203.6	1136.0	1273.0
East Twin Glacier	RGI60-01.23663	1321.0		1028.7	1190.5	1048.5
Echo Glacier	RGI60-01.01525	1299.7	1323.6	1231.5	1225.2	
Field Glacier	RGI60-01.01520	1369.8	1448.7	1389.3	1467.1	1483.5
Gilkey Glacier	RGI60-01.00704	1704.1	1422.8	1466.8	1462.2	1541.4
Herbert Glacier	RGI60-01.01245	1478.1	1113.4	1058.3	1072.7	1312.6
Laughton Glacier	RGI60-01.00660					1053.9
Lemon Creek Glacier	RGI60-01.01104		1143.7	1133.0		1196.1
Llewellyn Glacier	RGI60-01.23662	1722.1	1700.6	1682.5	1729.3	1710.2
Meade Glacier	RGI60-01.01524	1672.3	1526.5	1411.6	1472.9	1547.1
Mendenhall Glacier	RGI60-01.00709	1468.5	1092.0	996.1		1173.4
Norris Glacier	RGI60-01.01470	1365.7	1037.6	977.0		1051.5
Ogive Glacier	RGI60-01.01153	1579.2	1521.3	1537.8	1359.8	1469.0
Schubee Glacier	RGI60-01.00780					1380.9
South Glacier	RGI60-01.01401	1685.8	1510.2	1602.7	1647.3	1657.3
Taku Glacier	RGI60-01.01390	1444.8	1181.4	1016.2	882.4	1028.5
Thiel Glacier	RGI60-01.01242	1463.5	1191.3	1143.2	1127.0	
Tulsequah Glacier	RGI60-01.01521	1683.2	1499.8	1434.5	1496.2	1542.3
Warm Creek Glacier	RGI60-01.01306	1741.4	1569.4	1532.4	1558.4	1677.9
West Twin Glacier	RGI60-01.01514	1398.5	1199.2	1220.6	1293.6	1089.2
Willison Glacier	RGI60-01.01513	1776.7	1695.8	1664.0	1764.6	1719.0
All glaciers	Count	178	178	168	253	245
	Mean	1611.9	1444.3	1415.8	1518.3	1501.7
	Median	1636.6	1471.2	1456.9	1562.0	1557.2
	Standard Deviation	161.5	222.8	248.2	245.3	238.8

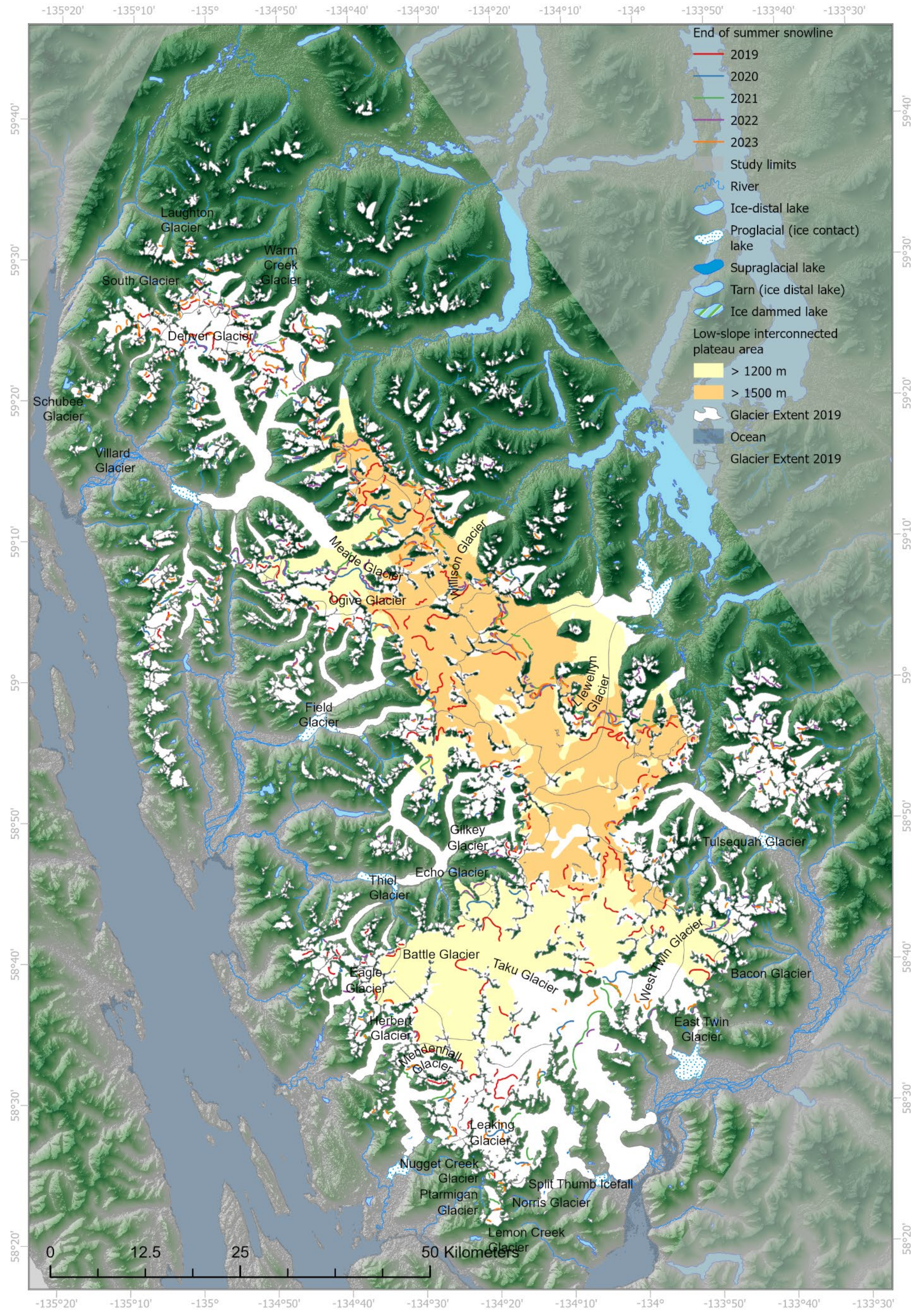

Supplementary figure 13. End of summer snowlines mapped from Sentinel imagery across Juneau Icefield, 2019-2023. Overlain on ASTER GDEM, the Global Digital Elevation Model produced by ASTER, courtesy of NASA/JPL-Caltech.

Supplementary figure 14a shows the elevations of the icefield wide mapped end-of-summer snowlines in comparison with mapped snowlines from Taku Glacier and Lemon Creek Glacier from 1946-2023 (Supplementary figure 14b), using an updated dataset from Pelto (2019)[38], and measured equilibrium line altitude for these two glaciers over the same time period, using data derived from the USGS Benchmark Glacier Program[4]; see also reference publications[26,39] (Supplementary figure 14c). These datasets show that, although the year 2019 was an unusually high year for snowline elevation, snowlines are reaching the plateau frequently, with both snowlines and ELAs occurring above the plateau height of 1200 m since 2005.

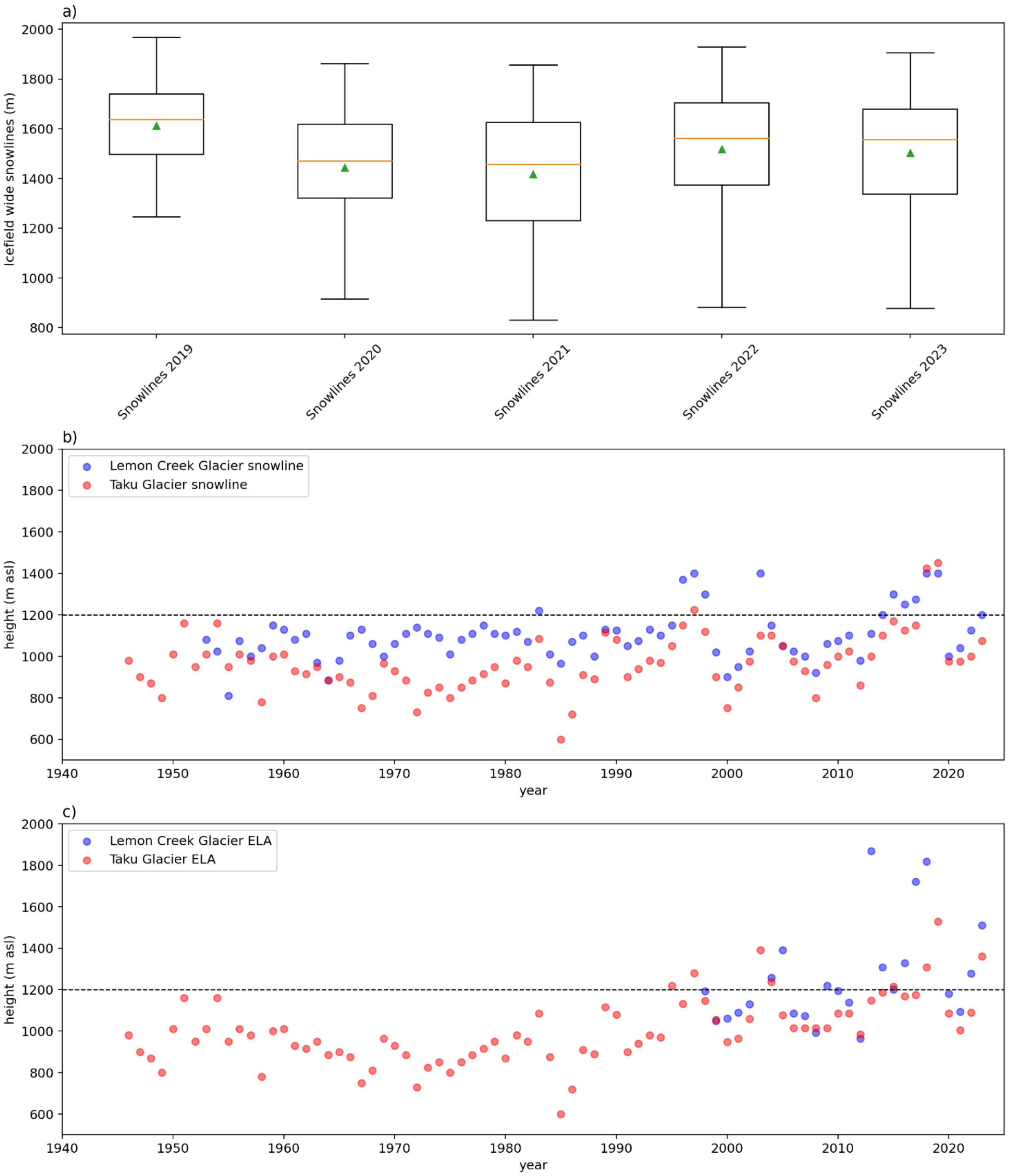

Supplementary figure 14. Snowlines and ELAs for Juneau Icefield. a) Icefield wide mapped snowlines, from late summer Sentinel imagery, 2019 to 2023. b) Snowlines for Taku Glacier and Lemon Creek Glacier, 1940-2022, mapped annually and manually from Landsat imagery. Updated dataset from Pelto (2019)[38]. The Taku Glacier plateau at 1200 m asl is shown with a dashed line. c) Glacier equilibrium line altitude (ELA) measurements for Taku Glacier and Lemon Creek Glacier from 1946-2023, derived from the USGS Benchmark Glacier Program[4]; see also reference publications[26,39]. The Taku Glacier plateau at 1200 m asl is shown with a dashed line.

5.7 Albedo

5.7.1 All sensors

We tested whether rising snowlines and glacier recession and fragmentation impacted glacier albedo by calculating average albedo across the icefield using a stack of all available Landsat satellites 5, 7, 8 and 9 (LT05, LE07, LC08 and LC09 respectively) images for the years 1987-2023 in Google Earth Engine[40], using a limit of 20% cloud cover over land from the USGS Metadata. We calculated average albedo within the 1990, 2005 and 2019 glacier extents to explore the variations due to glacier recession and fragmentation as well as to darkening of the glacier and snow surface. Our full stack included 299 images (Supplementary table 15), with Landsat 7 (LE07) being most frequently included and over the longest timeframe.

Supplementary table 15. Summary of the albedo stack

Summary statistics	Satellite	Sensor	Count	First scene	Last scene
Full stack			299	Jun-86	Aug-23
LT05	Landsat 5	EM	80	Jun-86	May-11
LE07	Landsat 7	ETM+	133	Aug-99	Jul-23
LC08	Landsat 8	OLI	76	Jun-13	Aug-23
LC09	Landsat 9	OLI	10	Feb-22	Aug-23

Using all available sensors from 1987-2023 but selecting only for scenes acquired in July, August and September (the months with highest ablation and highest snowlines) yielded an icefield wide average albedo of 0.78 ± 0.03 (95% confidence interval) when the 2019 glacier outlines were used, with a mean of 0.86 ± 0.03 on the plateau. Using the larger 1990 glacier outlines yielded a mean of 0.75 ± 0.03 (Supplementary table 16). Icefield wide mean albedos therefore rise when clipped to the 1990, 2005, 2019 or plateau outlines respectively, due to the inclusion of more exposed rock and hillslopes in the older outlines as glaciers recede and fragment over the study period. The plateau has the highest summer albedo, reflecting the high percentage of snow on this icefield accumulation area.

Supplementary figure 15 shows icefield-wide albedos for all sensors (a, b) and also only for Landsat 7 (c, d), across the two time periods (1987-2009 and 2010-2023). Decreases in albedo between the two periods were observed in all seasons, but were particularly pronounced in the summer and autumn seasons (Supplementary figure 15b).

Supplementary table 16. Icefield wide average albedos, calculated for the icefield as of 1990, 2005 and 2019, and also for the plateau area (above 1500 m in the Copernicus DEM).

		Summer means (July, August, September)			
		**1990 glacier outlines**	**2005 glacier outlines**	**2019 glacier outlines**	**Plateau (above 1500 m)**
**All sensors**					
**1987-2023**	Mean	0.75	0.78	0.79	0.86
	Confidence interval	0.03	0.03	0.03	0.03
	Standard deviation	0.01	0.01	0.01	0.01
	Median	0.78	0.81	0.82	0.91
	Minimum	0.42	0.44	0.44	0.55
	n	90	90	90	90
**1987-2009**	Mean	0.81	0.84	0.85	0.92
	Confidence interval	0.03	0.02	0.02	0.02
	Standard deviation	0.09	0.08	0.08	0.06
	Median	0.82	0.84	0.85	0.94
	Minimum	0.56	0.60	0.61	0.71
	n	50	50	50	50
**2010-2023**	Mean	0.67	0.70	0.71	0.78
	Confidence interval	0.03	0.04	0.04	0.04
	Standard deviation	0.14	0.14	0.14	0.14
	Median	0.64	0.66	0.67	0.74
	Minimum	0.42	0.44	0.44	0.55
	n	40	40	40	40

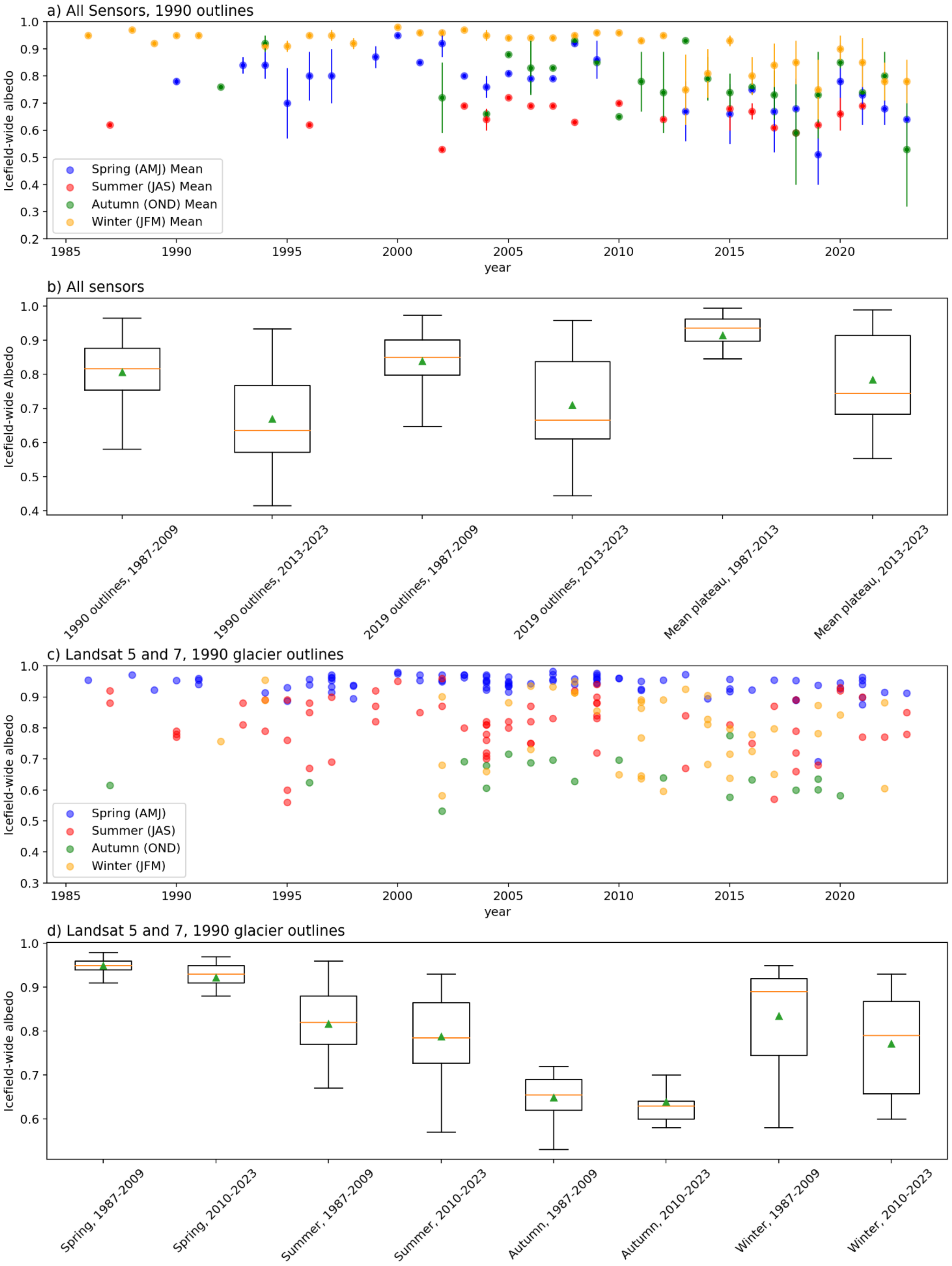

Supplementary figure 15. Icefield-wide albedo, using 1990 glacier outlines, in different seasons. Mean and standard deviation is shown. A) Icefield wide albedo using all sensors, with seasonal means and standard deviations shown. b) Icefield-wide albedo, for different glacier areas, showing the difference between two time periods: 1987-2009, and 2010-2023. C) As (a), but using only the Landsat 7 sensor. d) Icefield-wide albedos derived only from Landsat 7, showing seasonal differences and two time periods: 1987-2009, and 2009-2023. 1990 glacier outlines were used.

Supplementary table 17. Seasonal means for albedo across Juneau Icefield, using 1990 glacier outlines and all available sensors.

	Mean albedo				Standard deviation			
Month	JFM	AMJ	JAS	OND	JFM	AMJ	JAS	OND
Year	Spring	Summer	Autumn	Winter	Spring	Summer	Autumn	Winter
1986	0.95				0.00			
1987			0.62				0.00	
1988	0.97				0.00			
1989	0.92				0.00			
1990	0.95	0.78			0.00	0.01		
1991	0.95				0.01			
1992				0.76				0.00
1993		0.84				0.03		
1994	0.91	0.84		0.92	0.00	0.05		0.03
1995	0.91	0.70			0.02	0.13		
1996	0.95	0.80	0.62		0.01	0.09	0.00	
1997	0.95	0.80			0.02	0.10		
1998	0.92				0.02			
1999		0.87				0.04		
2000	0.98	0.95			0.00	0.00		
2001	0.96	0.85			0.01	0.00		
2002	0.96	0.92	0.53	0.72	0.01	0.05	0.00	0.13
2003	0.97	0.80	0.69		0.00	0.00	0.00	
2004	0.95	0.76	0.64	0.66	0.02	0.04	0.04	0.00
2005	0.94	0.81	0.72	0.88	0.01	0.01	0.00	0.00
2006	0.94	0.79	0.69	0.83	0.00	0.05	0.00	0.10
2007	0.94	0.79	0.69	0.83	0.01	0.00	0.00	0.00
2008	0.95	0.92	0.63	0.93	0.01	0.00	0.00	0.02
2009	0.96	0.86		0.85	0.01	0.07		0.00
2010	0.96		0.70	0.65	0.00		0.00	0.00
2011	0.93			0.78	0.01			0.11
2012	0.95		0.64	0.74	0.00		0.00	0.15
2013	0.75	0.67		0.93	0.13	0.11		0.00
2014	0.81			0.79	0.09			0.08
2015	0.93	0.66	0.68	0.74	0.02	0.11	0.08	0.07
2016	0.80	0.75	0.67	0.76	0.07	0.00	0.03	0.03
2017	0.84	0.67	0.61	0.73	0.08	0.15	0.00	0.09
2018	0.85	0.68	0.59	0.59	0.08	0.11	0.01	0.19
2019	0.75	0.51	0.62	0.73	0.11	0.11	0.02	0.16
2020	0.90	0.78	0.66	0.85	0.05	0.14	0.06	0.02
2021	0.85	0.73	0.69	0.74	0.09	0.11	0.00	0.00
2022	0.78	0.68		0.80	0.07	0.06		0.09
2023	0.78	0.64		0.53	0.08	0.13		0.21
Mean 1986-2009	0.95	0.83	0.65	0.82	0.01	0.04	0.00	0.03
mean 2010-2023	0.85	0.68	0.65	0.74	0.06	0.10	0.02	0.09

5.7.2 Landsat 5 and 7 only

In order to investigate whether differences in sensor contributed to this decrease in albedo, we calculated change in albedo over time using just Landsat 7 (LE07) data from all seasons (Supplementary table 18) and Landsat 5 and 7 data (Supplementary figure 15c, d). For just Landsat 7 alone, the icefield wide albedo within the 1990 glacier outlines was also lower from 2010-2023 (mean = 0.81, standard deviation = 0.1) than from 1999-2009 (mean = 0.87, standard deviation = 0.02); t (131) = 2.76, $p < 0.05$.

This result was replicated when including the Landsat 5 sensor as well, with lower albedos from 2010-2023 (mean = 0.81, standard deviation = 0.11) than from 1987-2009 (mean = 0.86, standard deviation = 0.11); t (211) = 3.28, $p < 0.05$. When including the longer time series provided by both Landsat 5 and 7, the difference was greatest in the summer season, though albedos decreased in all seasons (Supplementary figure 15d). Decreases in albedo in autumn and winter may reflect an observed shortening of the ablation season and lengthening of the accumulation season[41].

Supplementary table 18. Icefield-wide albedo, using only Landsat 7 sensor LE07, for both the summer months (July, August, September) and the entire stack.

		1990 glacier outlines	2005 glacier outlines	2019 glacier outlines	Plateau (above 1500 m)
			**Summer means (JAS)**		
1999-2022	Mean	0.81	0.84	0.85	0.93
	Confidence interval	0.03	0.03	0.03	0.02
	Standard deviation	0.09	0.08	0.08	0.06
	Median	0.82	0.85	0.85	0.95
	Minimum	0.57	0.61	0.61	0.70
	n	35	35	35	35
			**Whole stack**		
1999-2009	Mean	0.87	0.89	0.90	0.93
	Confidence interval	0.03	0.03	0.03	0.03
	Standard deviation	0.12	0.11	0.11	0.10
	Median	0.93	0.95	0.96	0.97
	Minimum	0.53	0.56	0.57	0.62
	n	58	58	58	58
2010-2023	Mean	0.81	0.84	0.85	0.89
	Confidence interval	0.03	0.03	0.03	0.02
	Standard deviation	0.12	0.12	0.12	0.11
	Median	0.84	0.89	0.90	0.92
	Minimum	0.57	0.61	0.61	0.62
	n	75	75	75	75

Any use of trade, firm, or product names is for descriptive purposes only and does not imply endorsement by the U.S. Government.

6 REFERENCES

1 National Oceanic and Atmospheric Administration (NOAA). *Climate at a Glance: City Time Series*, <https://www.ncei.noaa.gov/access/monitoring/climate-at-a-glance/> (2021).

2 Masson-Delmotte, V. *et al.* Climate change 2021: the physical science basis. *Contribution of working group I to the sixth assessment report of the intergovernmental panel on climate change* **2** (2021).

3 Millan, R., Mouginot, J., Rabatel, A. & Morlighem, M. Ice velocity and thickness of the world's glaciers. *Nature Geoscience* (2022). https://doi.org:https://doi.org/10.1038/s41561-021-00885-z

4 U.S. Geological Survey, B. G. P. Glacier-wide mass balance and compiled data inputs (ver. 8.0, November 2023): U.S. Geological Survey data release. (2016).

5 Hersbach, H. *et al.* The ERA5 global reanalysis. *Quarterly Journal of the Royal Meteorological Society* **146**, 1999-2049 (2020). https://doi.org:https://doi.org/10.1002/qj.3803

6 Gaglioti, B. V. *et al.* Timing and potential causes of 19th-century glacier advances in coastal Alaska based on tree-ring dating and historical accounts. *Frontiers in Earth Science* **7**, 82-82 (2019).

7 Wiles, G. C. *et al.* Surface air temperature variability reconstructed with tree rings for the Gulf of Alaska over the past 1200 years. *The Holocene* **24**, 198-208 (2014).

8 Forbes, V., Ledger, P. M., Cretu, D. & Elias, S. A sub-centennial, Little Ice Age climate reconstruction using beetle subfossil data from Nunalleq, southwestern Alaska. *Quaternary International* **549**, 118-129 (2020). https://doi.org:https://doi.org/10.1016/j.quaint.2019.07.011

9 Winski, D. *et al.* A 400-Year Ice Core Melt Layer Record of Summertime Warming in the Alaska Range. *Journal of Geophysical Research: Atmospheres* **123**, 3594-3611 (2018). https://doi.org:https://doi.org/10.1002/2017JD027539

10 Hu, F. S., Ito, E., Brown, T. A., Curry, B. B. & Engstrom, D. R. Pronounced climatic variations in Alaska during the last two millennia. *Proceedings of the National Academy of Sciences* **98**, 10552-10556 (2001).

11 Loso, M. G. Summer temperatures during the Medieval Warm Period and Little Ice Age inferred from varved proglacial lake sediments in southern Alaska. *Journal of Paleolimnology* **41**, 117-117 (2009).

12 Porter, S. C. in *Encyclopedia of Quaternary Science (Second Edition)* (eds Scott A. Elias & Cary J. B. T. Mock) 269-276 (Elsevier, 2013).

13 Wiles, G. C., Barclay, D. J. & Calkin, P. E. Tree-ring-dated 'Little Ice Age' histories of maritime glaciers from western Prince William Sound, Alaska. *The Holocene* **9**, 163-173 (1999). https://doi.org:doi:10.1191/095968399671927145

14 Koch, J. & Clague, J. J. Extensive glaciers in northwest North America during Medieval time. *Climatic Change* **107**, 593-613 (2011). https://doi.org:10.1007/s10584-010-0016-2

15 Barclay, D. J., Wiles, G. C. & Calkin, P. E. Holocene glacier fluctuations in Alaska. *Quaternary Science Reviews* **28**, 2034-2048 (2009).

16 Molnia, B. F. Late nineteenth to early twenty-first century behavior of Alaskan glaciers as indicators of changing regional climate. *Global and Planetary Change* **56**, 23-56 (2007).

17 Lawrence, D. B. Glacier fluctuation for six centuries in southeastern Alaska and its relation to solar activity. *Geographical Review* **40**, 191-223 (1950).

18 Miller, M. M. Inventory of Terminal Position Changes in Alaskan Coastal Glaciers Since the 1750's. *Proceedings of the American Philosophical Society* **108**, 257-273 (1964).

19 Wentworth, C. K. & Ray, L. L. Studies of certain Alaskan glaciers in 1931. *Bulletin of the Geological Society of America* **47**, 879-934 (1936).

20 Knopf, A. The Eagle River region, southeastern Alaska: US Geol. *Survey Bull* **502**, 36-40 (1912).

21 Röthlisberger, F. 1986: 10,000 Jahre Gletschergeschichte der Erde. Aarau: Sauerlander. (1986).

22 Pelto, M., Kavanaugh, J. & McNeil, C. Juneau Icefield mass balance program 1946–2011. *Earth System Science Data* **5**, 319-330 (2013).

23 Davies, B. *et al.* Topographic controls on ice flow and recession for Juneau Icefield (Alaska/British Columbia). *Earth Surface Processes and Landforms* **47**, 2357-2390 (2022). https://doi.org:https://doi.org/10.1002/esp.5383
24 Clague, J. J., Koch, J. & Geertsema, M. Expansion of outlet glaciers of the Juneau Icefield in northwest British Columbia during the past two millennia. *The Holocene* **20**, 447-461 (2010). https://doi.org:doi:10.1177/0959683609353433
25 Motyka, R. J. Little Ice Age subsidence and post Little Ice Age uplift at Juneau, Alaska, inferred from dendrochronology and geomorphology. *Quaternary Research* **59**, 300-309 (2003). https://doi.org:http://dx.doi.org/10.1016/S0033-5894(03)00032-2
26 McNeil, C. *et al.* Explaining mass balance and retreat dichotomies at Taku and Lemon Creek Glaciers, Alaska. *Journal of Glaciology* **66**, 530-542 (2020). https://doi.org:10.1017/jog.2020.22
27 Motyka, R. J. & Begét, J. E. Taku Glacier, Southeast Alaska, U.S.A.: Late Holocene History of a Tidewater Glacier. *Arctic and Alpine Research* **28**, 42-51 (1996). https://doi.org:10.2307/1552084
28 Motyka, R. J. Taku Glacier Advance: preliminary analysis. 32 (Alaska Division of Geological and Geophysical Surveys, Juneau, Alaska 99801, 1989).
29 Gilmore, R. A. Descriptive report: Southeast Alaska, Taku River to Flat Point. Register Number 8032. (U.S. Coast and Geodetic Survey, 1953).
30 Pelto, M. S. *et al.* The equilibrium flow and mass balance of the Taku Glacier, Alaska 1950–2006. *The Cryosphere* **2**, 147-157 (2008). https://doi.org:10.5194/tc-2-147-2008
31 Heusser, C. J. & Marcus, M. G. Historical variations of Lemon Creek Glacier, Alaska, and their relationship to the climatic record. *Journal of Glaciology* **5**, 77-86 (1964).
32 Sikorski, J. J., Kaufman, D. S., Manley, W. F. & Nolan, M. Glacial-Geologic Evidence for Decreased Precipitation During The Little Ice Age in The Brooks Range, Alaska. *Arctic, Antarctic, and Alpine Research* **41**, 138-150 (2009). https://doi.org:10.1657/1523-0430-41.1.138
33 McNeil, C. *et al.* The Imminent Calving Retreat of Taku Glacier. *Eos, American Geophysical Union* (2021).
34 Hartmann, B. & Wendler, G. The Significance of the 1976 Pacific Climate Shift in the Climatology of Alaska. *Journal of Climate* **18**, 4824-4839 (2005). https://doi.org:10.1175/JCLI3532.1
35 Kienholz, C., Herreid, S., Rich, J. L., Arendt, A. A., Hock, R. & Burgess, E. W. Derivation and analysis of a complete modern-date glacier inventory for Alaska and northwest Canada. *Journal of Glaciology* **61**, 403-420 (2015).
36 Boyce, E. S., Motyka, R. J. & Truffer, M. Flotation and retreat of a lake-calving terminus, Mendenhall Glacier, southeast Alaska, USA. *Journal of Glaciology* **53**, 211-224 (2007).
37 Motyka, R. J., O'Neel, S., Connor, C. L. & Echelmeyer, K. A. Twentieth century thinning of Mendenhall Glacier, Alaska, and its relationship to climate, lake calving, and glacier run-off. *Global and Planetary Change* **35**, 93-112 (2002).
38 Pelto, M. Exceptionally High 2018 Equilibrium Line Altitude on Taku Glacier, Alaska. *Remote Sensing* **11**, 2378-2378 (2019).
39 O'Neel, S. *et al.* Reanalysis of the US Geological Survey Benchmark Glaciers: long-term insight into climate forcing of glacier mass balance. *Journal of Glaciology* **65**, 850-866 (2019). https://doi.org:10.1017/jog.2019.66
40 Gorelick, N., Hancher, M., Dixon, M., Ilyushchenko, S., Thau, D. & Moore, R. Google Earth Engine: Planetary-scale geospatial analysis for everyone. *Remote Sensing of Environment* **202**, 18-27 (2017). https://doi.org:https://doi.org/10.1016/j.rse.2017.06.031
41 Thoman, R. & Walsh, J. E. *Alaska's Changing Environment: Documenting Alaska's Physical and Biological Changes Through Observations*. (International Arctic Research Center, University of Alaska Fairbanks, 2019).
